# Enhancing prime editor activity by directed protein evolution in yeast

**DOI:** 10.1038/s41467-024-46107-z

**Published:** 2024-03-07

**Authors:** Yanik Weber, Desirée Böck, Anastasia Ivașcu, Nicolas Mathis, Tanja Rothgangl, Eleonora I. Ioannidi, Alex C. Blaudt, Lisa Tidecks, Máté Vadovics, Hiromi Muramatsu, Andreas Reichmuth, Kim F. Marquart, Lucas Kissling, Norbert Pardi, Martin Jinek, Gerald Schwank

**Affiliations:** 1 Institute of Pharmacology and Toxicology, University of Zurich, Zurich, Switzerland; 2 Department of Biochemistry, University of Zurich, Zurich, Switzerland; 3 Department of Microbiology, Perelman School of Medicine, University of Pennsylvania, Philadelphia, PA USA; 4 Institute of Molecular Health Sciences, ETH Zurich, Zurich, Switzerland

**Keywords:** CRISPR-Cas9 genome editing, Molecular medicine, Targeted gene repair, CRISPR-Cas9 genome editing

## Abstract

Prime editing is a highly versatile genome editing technology that enables the introduction of base substitutions, insertions, and deletions. However, compared to traditional Cas9 nucleases prime editors (PEs) are less active. In this study we use OrthoRep, a yeast-based platform for directed protein evolution, to enhance the editing efficiency of PEs. After several rounds of evolution with increased selection pressure, we identify multiple mutations that have a positive effect on PE activity in yeast cells and in biochemical assays. Combining the two most effective mutations – the A259D amino acid substitution in nCas9 and the K445T substitution in M-MLV RT – results in the variant PE_Y18. Delivery of PE_Y18, encoded on DNA, mRNA or as a ribonucleoprotein complex into mammalian cell lines increases editing rates up to 3.5-fold compared to PEmax. In addition, PE_Y18 supports higher prime editing rates when delivered in vivo into the liver or brain. Our study demonstrates proof-of-concept for the application of OrthoRep to optimize genome editing tools in eukaryotic cells.

## Abstract (web summary)

Compared to traditional Cas9 nucleases prime editors (PEs) are less active. Here the authors use OrthoRep, a yeast-based platform for directed protein evolution to enhance the editing efficiency of PEs: they identify mutations that have a positive effect on kinetics and use this knowledge to generate an efficient in vivo PE.

## Introduction

Prime editors (PEs) can write genetic information in the genome when administered together with a prime editing guide RNA (pegRNA). PEs comprise an *Sp*Cas9 nickase (H840A – hereafter referred to as nCas9) fused to an engineered reverse transcriptase (RT) derived from Moloney Murine leukemia virus (M-MLV). pegRNAs consist of a Cas9 guide RNA, fused at the 3’ end to a reverse transcriptase template (RTT) sequence and a primer binding site (PBS). Upon binding of the PE ribonucleoprotein (RNP) complex to the genomic target site it initiates a nick at the non-target strand (i.e. the DNA strand that is not targeted by the guide RNA). This strand anneals to the complementary PBS, which primes reverse transcription of the RTT segment of the pegRNA to generate a DNA flap. Cellular DNA repair facilitates integration and copying of the flap, leading to the installation of the edit^1^.

Despite the high versatility of PEs, they are comparably less efficient than classical Cas9 nucleases, which induce DNA double strand breaks, or base editors (BEs), which modify DNA via deamination^2^. Recent studies therefore utilized rational design to increase the performance of prime editing. For example, pegRNAs have been protected from exonucleases by fusing structural motifs to the 3’ end (epegRNAs)^3^, the cellular DNA mismatch repair machinery has been altered to favor selection of the introduced edit^4^, and codon usage as well as domain architecture of the PE have been optimized (PEmax)^4^. Furthermore, a recent study employed directed protein evolution using phage-assisted continuous evolution (PACE) to enhance the activity of different RT enzymes for prime editing^5^.

In this study we employed Orthogonal DNA replication (OrthoRep)^6^, a protein evolution approach in yeast, to increase the activity of PEs in a eukaryotic environment. PEmax variants resulting from four rounds of evolution showed increased activity in yeast cells, and in vitro in a biochemical assay. Recombining the mutations of the two best-performing variants resulted in the variant PE_Y18, which contains amino acid substitutions A259D in nCas9 and K445T in the M-MLV RT. PE_Y18 shows enhanced activity in cell lines when delivered as a plasmid, mRNA or RNP complex. Furthermore, PE_Y18 exhibited increased activity in the mouse brain and the liver upon AVV-mediated delivery.

## Results

### Development of an OrthoRep selection logic for PE evolution

**Fig. 1:**
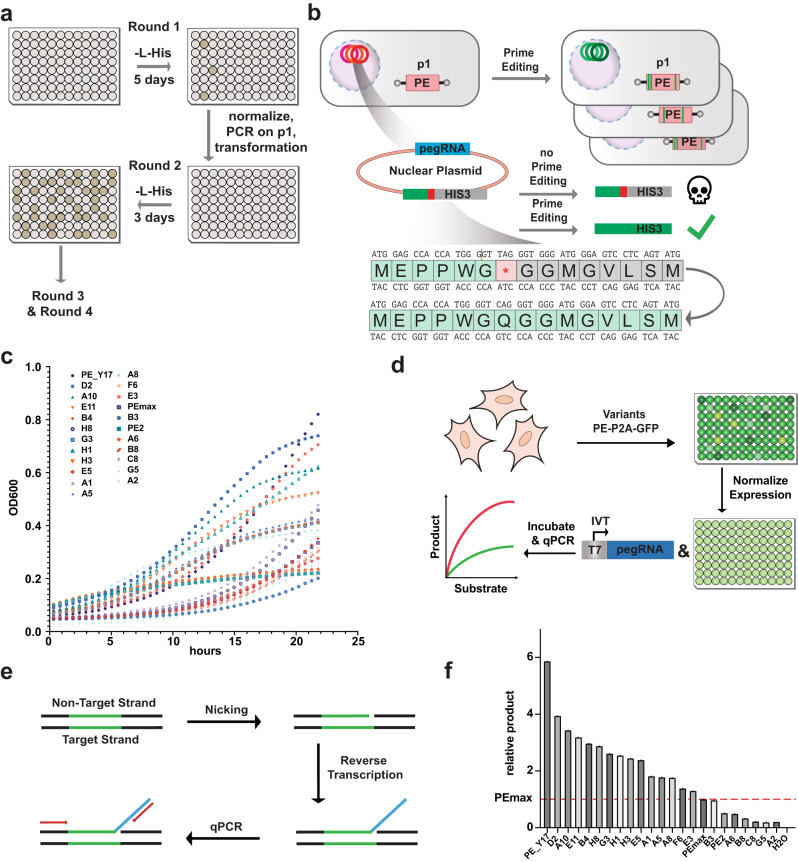
Evolution of PE variants with enhanced activity in OrthoRep. **a** Parallel evolution of prime editors (PE) by culturing yeast cells in 96 well plates over four subsequent rounds in L-histidine-depleted (-L-His) selection media. In the first round of evolution, outgrowing yeast cells were normalized prior to extraction of the linear plasmid (p1) via PCR and transformation into fresh host cells for the second round. The same procedure was repeated for the third and fourth round. **b** PE variants containing mutations that increase prime editing rates are enriched in selective conditions: a stop codon in front of the auxotrophic marker Imidazoleglycerol-phosphate dehydratase (HIS3) must be repaired by prime editing on the nuclear plasmid co-expressing the prime editing guide RNA (pegRNA) for successful yeast growth in selective conditions. **c** Effect of evolved PE variants on yeast growth quantified as optical density at 600 nm (OD600) under selective conditions. The assessed variants contained the following mutations: PE_Y17 (nCas9 S219A, RT K445T), D2 (nCas9 A259D), A10 (RT Y64W, K373R, R389C, L432M, K445T), E11 (RT Y64W), B4 (RT R44H), H8 (nCas9 S219A, RT K445T), G3 (nCas9 A259D, linker G30S, RT R389C, S606A), H1 (nCas9 A259D, RT Y64W), H3 (nCas9 A259D) E5 (nCas9 S219A, RT K445T), A1 (nCas9 A259D, linker G30S, RT R389C, S606A), A5 (nCas9 S219A, RT Y64C, K445T), A8 (nCas9 S219A, linker G30S, RT K445T), F6 (nCas9 A259D, RT R44H), E3 (nCas9 S320R), B3 (nCas9 R71C, A259D, linker G30S, RT K445T), A6 (nCas9 S318N, RT R389C, L432M), B8 (nCas9 S320N,RT K373R), C8 (nCas9 Y132C), G5 (nCas9 A259D,RT K373R), A2 (nCas9 S219A, RT K373R, K445T). Data are displayed as the mean of two individual replicates. **d** Schematic of the in vitro assay to assess PE kinetics (PEKIN). PE variants are subcloned as self-cleaving peptide fused to a green fluorescent protein (P2A-GFP) and transfected into HEK293T cells. Cells are lysed and PE expression levels are normalized by fluorescence intensity. An in vitro transcribed (IVT) pegRNA forms the RNP with the PE, which is incubated with a synthetic dsDNA substrate. **e** Schematics illustrating the strategy used to quantify PE activity in PEKIN via quantitative PCR (qPCR). **f** Quantified prime editing activities of the 21 PE variants isolated after four rounds of evolutions in OrthoRep relative to PEmax. Product formation of the variants is relative to the product formed by PEmax and was performed as a single replicate (*n* = 1).

OrthoRep is a yeast-based directed evolution system that employs an orthogonal error-prone DNA polymerase to trigger hypermutations (10^−5^ per base per generation) on a linear plasmid (p1) while ensuring that the mutation rate of the host genome remains unaffected^6^. To develop an OrthoRep evolution approach for selecting PE variants with increased activity, we generated GYR333 yeast strains containing linear p1 plasmids expressing PE1^1^, PE2^1^, or PEmax^4^, respectively. These strains were co-transformed with a plasmid encoding for the orthogonal, error-prone DNA polymerase TP-DNAP1 (L477V, L640Y, I777K, W814N)^7^, resulting in hypermutation of the PE variants. After a five-day mutagenic drift period, yeast strains were further transformed with a multicopy nuclear plasmid expressing an inactive version of the essential auxotrophic marker gene HIS3 and a pegRNA that enables the conversion of the inactive HIS3 variant back into an active variant (Fig. 1a, b; Supplementary Fig. 1). This setup ensured that under selective conditions yeast growth was linked to prime editing rates.

To first validate our selection approach, we started with the evolution of PE1^1^. PE1 lacks the activity-enhancing mutations that are present in the M-MLV RT domain of PE2^1^ and PEmax^4^, leading to substantially lower editing rates^1^. Yeast cells were transformed with the different plasmids as described above and seeded in a 96 well plate. After a five-day growth period in L-histidine depleted medium we visually observed yeast outgrowth in 5% of the wells (5 of 96). Verifying functionality of the evolution logic, sanger sequencing of the HIS3 locus revealed 71% editing in yeast cells grown under selection as compared to 10% editing in control wells where yeast cells were grown in non-selective media (Supplementary Fig. 2). In addition, yeast cells not transfected with the pegRNA-encoding plasmid did not grow under selection (0 out of 96 wells). Finally, when we sequenced PE1 variants subcloned from yeast cells grown under selection, we identified two of the mutations that were previously reported in PE2, D200N and T330P (Supplementary Table 1).

Next, we employed the same experimental setup to evolve PE2. Yeast cells were transformed with the different OrthoRep plasmids for the PE selection logic and again cultured under selection for five days. PE2 variants were isolated from outgrowing cells via PCR extraction and subcloned prior to analysis by Sanger sequencing and Oxford Nanopore sequencing^8^ (Supplementary Tables 1 and 2). To assess whether these variants show higher prime editing activity than PE2, three clones were randomly chosen and subcloned into the pCMV-PE2 mammalian expression vector. Transfection into HEK293T cells indeed revealed an up to a 2.6-fold increase in editing efficiency for isolated variants as compared to PE2 (Supplementary Fig. 3).

### Evolution and in vitro characterization of PEmax variants

After validating the functionality of our prime editing selection logic in OrthoRep, we attempted to evolve PEmax. This PE variant has been established from PE2 and shows higher editing activity due to mammalian-optimized codon usage, improved domain architecture, and two mutations in the nickase domain of Cas9^4^. Yeast cells were transformed with the different plasmids for PEmax evolution following a protocol that again allows for a five-day mutagenic drift period. Subsequently, they were cultured under selective conditions in a 96-well plate for five days (Supplementary Table 3). Wells that showed visual outgrowth of yeast cells were normalized to an OD of 0.5, pooled, and subjected to PCR amplification prior to transformation into fresh host cells for the next round of evolution. Consistent with the hypothesis that more active PEmax variants outcompete other variants, we observed an increase in the number of wells with visual yeast growth already after three days in the second selection round (Supplementary Table 3). During the third round of evolution, we observed yeast growth in 94 of 96 wells after three days, indicating the need to further intensify the selection pressure. Therefore, we replaced the HIS3 selection cassette containing a stop mutation with a modified version, which requires the incorporation of a 501 bp segment into the HIS3 gene (Supplementary Table 4). While the stringent selection pressure of this cassette did not enable yeast growth when the original PEmax variant was transfected, PEmax variants isolated from the third round of evolution led to visible yeast growth in six out of 96 wells after five days (Supplementary Tables 3 and 5).

To verify increased activity of PE variants selected over the four rounds of evolution, 21 clones were extracted by PCR and cloned on the linear p1 plasmid. These plasmids were transformed into yeast cells containing the wild-type TP-DNAP1 polymerase^6^, the HIS3 selection cassette, and the respective pegRNA to repair the inactivating stop codon. Importantly, 16 out of the 21 evolved variants conveyed higher fitness to yeast growth in selective conditions as compared to PEmax (Fig. 1c).

To benchmark the activity of the evolved PEmax variants in mammalian cell-lysates, we next developed a Prime Editing KINetics assay (PEKIN), which quantifies the ability of a PE to nick a dsDNA and generate a DNA flap. In brief, the 21 extracted PEmax variants were cloned into a mammalian expression vector, C-terminally linked to a P2A-GFP fusion protein with self-cleavage capabilities^9^. After expression in HEK293T cells and cell lysis, PE protein levels were normalized via GFP fluorescence. Subsequently, PE variants were complexed with an in vitro transcribed pegRNA and incubated with a synthetic double-stranded DNA template (Fig. 1d). Nicking of the DNA template followed by the extension from the pegRNA RTT through reverse transcription was then quantified by qPCR (Fig. 1e). Notably, of the 21 tested variants, 15 showed substantially greater flap generation than PEmax (Fig. 1f), with a strong correlation to the activity increase observed in the yeast growth assay (Supplementary Fig. 4).

**Fig. 2:**
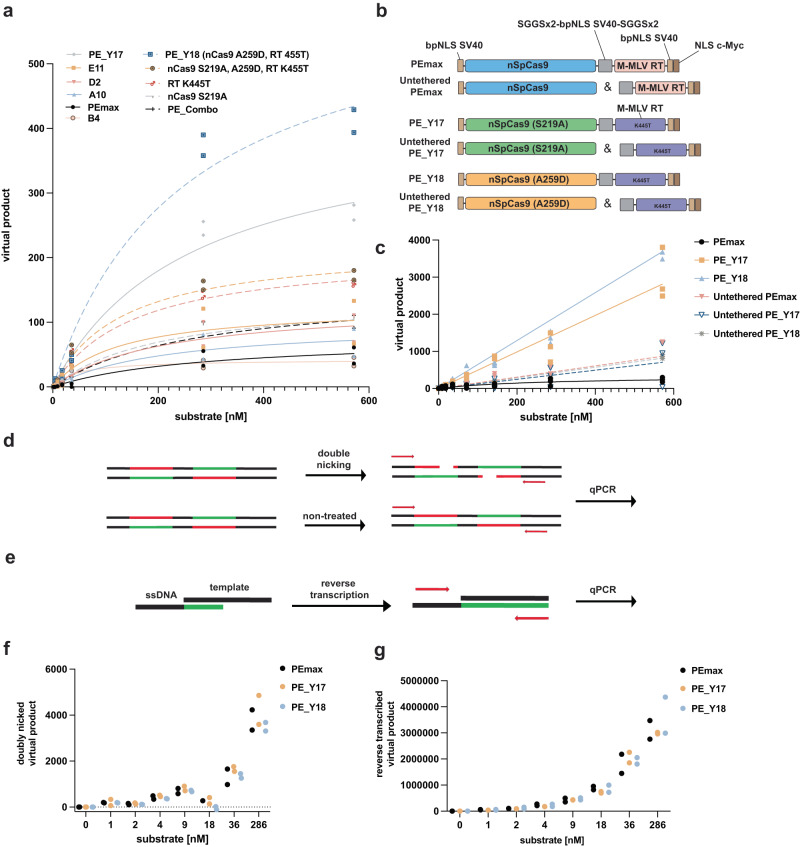
In vitro characterization of the activity of PE variants using cell lysates and purified proteins. **a** Quantification of PE activity with increasing concentrations of dsDNA substrate for PEmax (black), five selected PE variants obtained from OrthoRep (solid lines), and recombined versions of these variants (dashed lines). Individual values are represented from biological replicates (*n* = 2). **b** Schematic illustration of constructs that were characterized in PEKIN as purified proteins including the bipartite nuclear localisation signal from the siman virus 40 (bpSV40 NLS) and the nuclear localisation signal (NLS c-Myc) where indicated. PEmax was compared to the evolved variants PE_Y17 and PE_Y18 in a tethered and untethered form. **c** Quantification of prime editing rates of purified proteins in the PEKIN assay in the tethered (solid lines) and untethered form (dashed lines). Virtual products are calculated from respective Ct values of individual triplicates (*n* = 3). **d** Scheme of the assay used to determine nicking activity via double nicking of a synthetic DNA template. Red illustrates the non-target strand, whereas the target strand is illustrated in green. **e** Schematic of the adapted assay to disentangle reverse transcription activity on a single-stranded DNA (ssDNA) oligo via a complementary RNA template. **f** Quantification of ∆Ct of double nicked templates relative to the untreated quantified substrate concentrations. Experiments were performed as individual duplicates (*n* = 2). **g** Quantification of reverse transcribed product on a ssDNA oligo. Experiments were performed as individual duplicates (*n* = 2).

The five variants that exhibited greatest flap generation were further evaluated using a range of substrate concentrations (Fig. 2a). With the exception of B4, every variant demonstrated higher activity than PEmax across all concentrations. Subsequently, we explored the possibility of an additive effect by cumulating all mutations discovered in the top five variants in a single variant (PE_Combo; S219A, A259D in nCas9 and R44H, Y64W, K373R, R389C, L432M, K445T in the RT). However, the activity of PE_Combo was lower than the activity of PE_Y17, which was the most active variant selected by OrthoRep and contains a S219A amino acid change in nCas9 and a K445T amino acid change in the M-MLV-RT. Notably, PE_D2, the second best performing variant, only contained a mutation in nCas9 (A259D) but not in the RT, prompting us to generate a variant where we combined the A259D mutation in nCas9 with the RT K445T mutation of PE_Y17. Importantly, the resulting variant, termed PE_Y18, showed substantially higher activity than PE_Y17 (Fig. 2a).

To validate results obtained from normalized cell lysates, we next repeated the PEKIN assay with recombinantly expressed and purified PE_Y17, PE_Y18 and PEmax proteins (Supplementary Fig. 5). Confirming increased activity of PE_Y17 and PE_Y18, both variants led to increased flap formation compared to PEmax with all tested dsDNA substrate concentrations (Fig. 2c).

To gain a deeper insight into the functional role of the mutations in PE_Y17 and PE_Y18, we also introduced them into untethered PEmax, in which the nCas9 and M-MLV RT were not fused^10,11^. Interestingly, in this context we observed no increase in product formation compared to the control (untethered PEmax) (Fig. 2c). Therefore, we adapted the PEKIN assay to determine if either the nicking activity of SpCas9 or the reverse transcription activity of the M-MLV-RT are increased in full-length PE_Y17 and PE_Y18. To assess DNA nicking activity, we supplied the PE RNP with a guide RNA that targets a dsDNA template on both strands (Fig. 2d). To assess reverse transcription activity, we supplied the PE RNP with an ssDNA and an RNA containing a PBS for the ssDNA and an RTT template (Fig. 2e). Quantification of product formation by qPCR, however, revealed no increase in DNA nicking or reverse transcription activity of PE_Y17 or PE_Y18 compared to PEmax (Fig. 2f, g). Thus, our data indicates that the identified mutations do not directly influence the activity of the Cas9 nickase or the M-MLV RT, but instead enhance the activity of the PE fusion complex in a more complex manner, for example by enhanced priming of the PBS specifically within the R-loop.

### Validation of PE_Y17 and PE_Y18 in mammalian cells

**Fig. 3:**
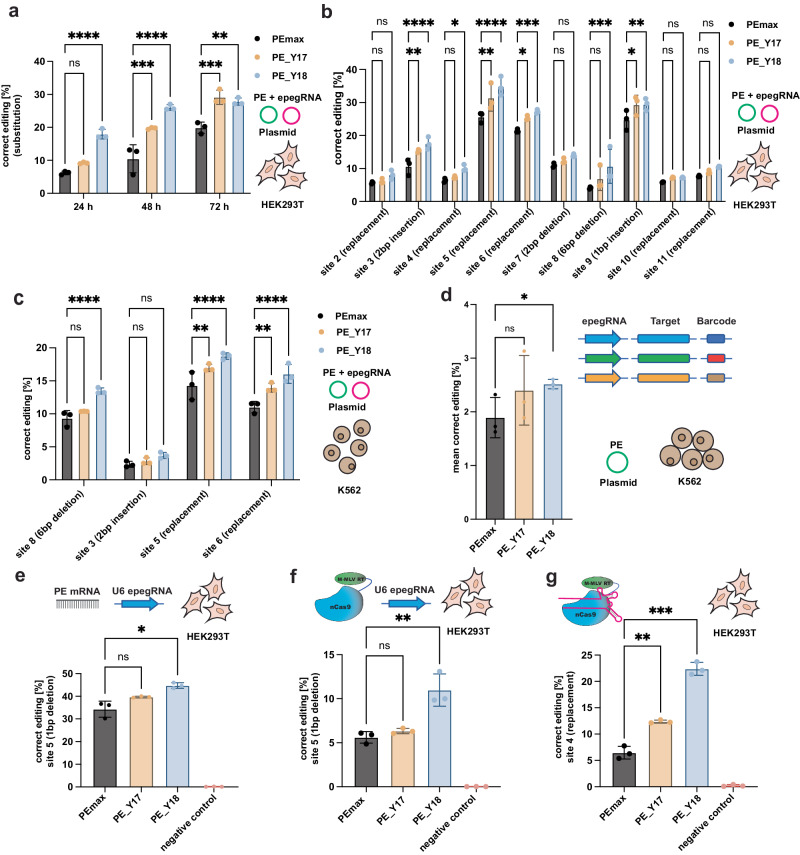
Comparison of editing rates with evolved PE variants in mammalian cell lines. **a** Correct percentage of substitutions assessed by deep sequencing in cells transfected with plasmids encoding for PEmax, PE_Y17, PE_Y18 together with plasmids encoding for the enhanced prime editor guide RNAs (epegRNAs) at different time points on site 1 in HEK293T cells; not significant (ns) *P* = 0.5476 ****P < 0.0001, ***P = 0.0002, ****P < 0.0001, ***P = 0.0002 and **P < 0.0012 (left to right). **b** Editing rates of PE_Y17, PE_Y18 and PEmax at other loci in HEK293T cells, analyzed 48 h after plasmid transfection; not significant (ns) *P* = 0.9462, ns *P* = 0.2578, **P = 0.0084, ****P < 0.0001, ns *P* = 0.8254, *P = 0.0478, **P = 0.001, ****P < 0.0001, *P = 0.0341, ***P = 0.0008, ns *P* = 0.6543, ns *P* = 0.1331, ns *P* = 0.1621, ***P = 0.0002, *P = 0.011, **P = 0.0086, ns *P* = 0.6507, ns *P* = 0.6442, ns *P* = 0.7257 and ns *P* = 0.1566 (left to right). **c** Editing rates of PEmax, PE_Y17 and PE_Y18 on endogenous loci in K562 cells, analyzed 120 h after transfection; not significant (ns) *P* = 0.3622, ****P < 0.0001, ns *P* = 0.8171, ns *P* = 0.2033, **P = 0.0049, ****P < 0.0001, **P = 0.0022 and ****P < 0.0001 (left to right). **d** Editing rates of PEmax, PE_Y17 and PE_Y18 on a self-targeting library in K562 cells. The self-targeting library encoding for epegRNAs and their respective target sites was integrated into cells using lentiviral vectors prior to PE plasmid transfection and analysis of editing rates by deep sequencing after 120 h; not significant (ns) *P* = 0.305 and *P < 0.0486 (left to right). **e** Editing rates with PE variants encoded on mRNA and nucleofected into HEK293T cell line expressing an epegRNA targeting site 12; not significant (ns) *P* = 0.1152 and *P = 0.0145 (left to right). **f** Nucleofection of PEmax, PE_Y17 or PE_Y18 protein into HEK293T cells expressing an epegRNA targeting site 12; not significant (ns) *P* = 0.6306 and **P = 0.0042 (left to right). **g** Nucleofection of PE RNPs with a chemically modified pegRNA targeting site 4 in HEK293T cells; **P = 0.0079, ***P = 0.0002 (left to right). Data are displayed as means±s.d. of three independent experiments (*n* = 3) and were analyzed using a two-way ANOVA using Tukey’s multiple comparisons.

To determine if the enhanced activity of evolved PEmax variants also leads to higher editing in mammalian cells, we first transfected plasmids expressing PE_Y17, PE_Y18, or PEmax together with an epegRNA that induces a C-to-T transition mutation at site 1 in HEK293T cells (Supplementary Table 6). After 24, 48 and 72 h cells were harvested and editing rates were assessed by NGS. At all three timepoints we observed substantially higher editing with PE_Y17 and PE_Y18 as compared to PEmax (on average 1.6-fold with PE_Y17 and 2.3-fold with PE_Y18; Fig. 3a), without detecting an increase in indel rates (Supplementary Fig. 6a). We then targeted additional loci in HEK293T and K562 cells with epegRNAs introducing substitutions, insertions, or deletions (Supplementary Table 6). Again, improved editing without enhanced indel rates was observed for PE_Y17 and PE_Y18, with an average 1.2-fold increase with PE_Y17 and 1.4-fold increase with PE_Y18 in HEK293T cells (Fig. 3b), and an average 1.2-fold increase with PE_Y17 and 1.4-fold increase with PE_Y18 in K562 cells (Fig. 3c). To further assess editing rates with PE_Y17 and PE_Y18 at a larger number of target sites, we generated a self-targeting epegRNA library containing 200 different sequences^12^. Lentiviral vectors containing epegRNA expression cassettes and the respective target sites were integrated into K562 cells prior to the transfection with PE variants. Deep sequencing of target sites again revealed a trend for increased editing with PE_Y17 and significantly increased editing with PE_Y18 (1.3-fold increase to PEmax; Fig. 3d). To next assess if the evolved PE variants lead to increased off-target editing, we targeted two loci with pegRNAs that have known off-target activity^1,13^. However, while on-target editing was increased at both sites, deep-sequencing of the off-target sites did not reveal elevated activity (Supplementary Fig. 7).

To characterize the evolved PEmax variants further, we next delivered in vitro transcribed nucleoside-modified mRNAs encoding for PEmax, PE_Y17 or PE_Y18 into HEK293T cells that constitutively expressed an epegRNA introducing a single base pair deletion in site 12. Confirming our results from plasmid transfections, we observed a trend for higher editing with PE_Y17 and significantly higher editing (1.3-fold) for PE_Y18 compared to PEmax (Fig. 3e). Similarly, nucleofection of the purified PE proteins instead of mRNA into the same cell line led to a trend for higher editing at site 12 with PE_Y17 and a significantly higher editing (2-fold) with PE_Y18 (Fig. 3f). Finally, electroporation of RNPs consisting of the PE complexed with a synthetic pegRNA targeting site 4 demonstrated significantly higher editing rates for both evolved variants (1.9-fold for PE_Y17 and 3.5-fold for PE_Y18; Fig. 3g).

### PE_Y18 enhances prime editing rates in vivo

**Fig. 4:**
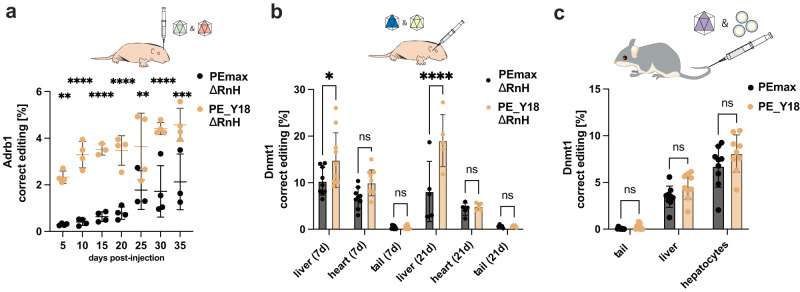
In vivo comparison of PE_Y18^∆RnH^ and PEmax delivered via AAV or mRNA-LNP. **a** Experimental setup and editing rates at the targeted Adrb1 locus in cortices. Intein-split PEmax^∆RnH^ and PE_Y18^∆RnH^ were packaged into AAV-PHP.eB capsids and injected intracerebroventricular into P1 mice. For PE_Y18^∆RnH^, four animals were individually treated for each timepoint. For PEmax, time points 5, 10, 15, 20, and 30 days were assessed by four (*n* = 4) individually treated animals, whereas three (*n* = 3) animals were treated for 25 and 35 days respectively; **P = 0.0012, ****P < 0.0001, ****P < 0.0001, ****P < 0.0001, **P = 0.0071, ****P < 0.0001 and ***P = 0.0003 (left to right). **b** Editing rates at the targeted Dnmt1 locus, assessed in different tissues at days 7 and 21 after temporal vein injection of the intein-split PE_Y18^∆RnH^ and PEmax^∆RnH^ packaged in AAV9. For PEmax^∆RnH^ nine animals (*n* = 9) were individually treated for the time point 7d and five animals (*n* = 5) for 21d respectively. For PE_Y18^∆RnH^ ten animals (*n* = 10) were treated for time point 7d and five (*n* = 5) for 21d respectively; *P = 0.0199, not significant (ns) *P* = 0.1939, ns *P* > 0.9999, ****P < 0.0001, ns *P* > 0.9999 and ns *P* > 0.9999 (left to right). **c** Four weeks before single administration of mRNA-LNPs (2 mg/kg PEmax or PE_Y18 mRNA) via the tail vein, male mice were injected with an scAAV9 expressing the epegRNA that targets the Dnmt1 locus. Editing rates after LNP administration were assessed in the tail, liver tissue, and isolated hepatocytes. Organs were analyzed from nine individually treated animals (*n* = 9) for PEmax and PE_Y18; not significant (ns) *P* = 0.999, ns *P* = 0.2932 and ns *P* = 0.1306 (left to right). Data are displayed as means±s.d. of the indicated and were analyzed using a two-way ANOVA using Tukey’s multiple comparisons.

Recently, prime editing approaches for introducing or correcting mutations in vivo in mice have been developed^5,10,11,14–19^. Therefore, we assessed if our most active variant, PE_Y18, also increases prime editing rates in different mouse tissues. Using adeno-associated viral (AAV) vectors, we first delivered PEmax and PE_Y18 into the murine brain. Both PE variants were expressed from the neuron-specific human synapsin 1 (hSyn1) promoter^20^, together with an epegRNA that installs a C-to-T transition mutation in the Adrb1 locus. Due to the limited packaging capacity of AAV^21^, they were also shortened by removing the RnaseH domain of the RT (PE_Y18^∆*RnH*^), and split into two parts using the intein-split systems as described previously^14,18^ (Supplementary Fig. 8). After packaging both vectors into AAV-PHP.eB^18^ capsids, they were injected into the ventricles in 1-day-old pups (P1). Importantly, deep sequencing of isolated brain tissues revealed significantly higher editing rates with PE_Y18^∆*RnH*^ compared to PEmax at all analyzed time points (5-35 days post injection; on average 4.7-fold increase; Fig. 4a). Furthermore,  we did not observe a significant increase in indel rates and PE expression levels were comparable between both variants (Supplementary Figs. 9a and 10a).

Next, we exchanged the epegRNA on the AAV vector with an epegRNA that installs a T-to-G transversion in the Dnmt1 locus, and the hSyn promoter with the ubiquitous CBh promoter. Vectors were packaged into AAV9 capsids, which enable transduction of various tissues including the liver and heart, and systemically delivered via the temporal vein into P1 mice. After 7- and 21-days, genomic DNA was isolated from the liver and heart and analyzed by NGS for editing rates. We observed significantly higher editing with PE_Y18^∆*RnH*^ in the liver (1.4-fold increase at day 7 and a 2.3-fold increase at day 21), and a statistically non-significant trend for higher editing in the heart (1.5-fold increase at day 7 and a 1.1-fold increase at day 21). Again, we did not observe a significant increase in indel rates with PE_Y18^∆*RnH*^, and expression levels were comparable between both variants (Fig. 4b; Supplementary Figs. 9b and 10b).

Finally, we benchmarked PEmax with PE_Y18 in a transient in vivo prime editing approach^19^, where nucleoside-modified mRNAs encoding for both PE variants were encapsulated into lipid nanoparticles (LNPs). Systemic delivery of 2 mg/kg mRNA-LNP into mice pre-treated with self-complementary AAV9 (scAAV9) encoding for the Dnmt1 epegRNA resulted in a 1.3-fold increase in editing rates with PE_Y18, which, nevertheless, was not statistically significant (*P* = 0.1306) (Fig. 4c).

## Discussion

In this study we demonstrate proof-of-concept for using OrthoRep to evolve PE variants with increased activity. After four rounds of evolutions, we isolated a number of PE variants exhibiting increased editing activity in yeast and higher DNA-flap generation in our in vitro PEKIN assay. The two most efficient PE variants, PE_Y17 and recombinant PE_Y18, also show higher activity at various target sites when delivered into cells. PE_Y18, moreover, led to higher prime editing rates in vivo in the brain and liver of mice. Interestingly, the activity-enhancing mutations did not show an effect when introduced into an untethered PE, where the nCas9 and M-MLV RT are not fused. This highlights the advantage of directly evolving full-length PEs instead of the individual components (nicking activity of nCas9 or reverse transcription activity of the RT). Since the linear plasmid p1 allows for encoding proteins with sizes up to 22 kb^22^, in principle even larger multi-component genome editing tools such as CRISPR-associated transposons^23,24^ could be evolved for higher activity in OrthoRep. Moreover, our selection approach to evolving PE variants is highly versatile, and stringency could be easily adapted. For example, instead of linking yeast growth to the installation of a single edit it could be linked to the requirement of installing several consecutive edits, either on the same or on different auxotrophic marker genes.

One drawback of our selection approach is the necessity to extract the PE variants after each selection round in order to retransform them into fresh host cells with unedited selection plasmids. Such manual intervention is not required during PACE^25^, where a phage carrying the genome editor infects fresh host cells with selection plasmids every 20 minutes. Nevertheless, while PACE has been successfully employed to optimize various genome editing tools^5,26,27^, selections occur in the bacterial cytoplasm. OrthoRep, in contrast, functions in eukaryotic cells, which has certain advantages. For example, it could be employed to screen for genome editors with more efficient nuclear import, or to evolve enzymes that require eukaryotic posttranslational modifications. Thus, it would be highly valuable to develop continuous selection approaches for genome editors in OrthoRep. This could be achieved by pairing the system with another editor that continuously reverts the essential mutation, or by incorporating abortive mating to continuously pass PE variants to fresh host cells.

Continuous PE evolution in OrthoRep could potentially lead to variants with higher activity than PE_Y18. This would be desired for the clinical translation of prime editing, since in vivo genome editing rates with PE_Y18 did not reach levels that are typically achieved with Cas9 nucleases or base editors^2^, and AAV doses above what would be deemed safe for human application^28,29^ had to be used. Another limitation for efficient prime editing is the stability of the pegRNA, particularly the 3’end, which is not protected by Cas9 and readily degraded by exo- and endonucleases. Future research should therefore not only be directed on the evolution of the PE, but also on refining pegRNA chemistry or design.

## Methods

### Ethical statement

Animal experiments were performed in accordance with protocols approved by the Kantonales Veterinäramt Zürich and in compliance with all relevant ethical regulations.

### Cloning

Plasmids containing the P1 landing pad pTBL1201_pUC_FDP, pTBL963_pcDNA3_1 and pAR-Ec633 (Addgene #130873) were gifts from Chang Liu. PE1 (pLYW118), PE2 (pLYE094) and PEmax (pLYW200) were integrated into pTBL1201_pUC_FDP using PCR amplification from psZ157 CRISPEY RT/Cas9 (Addgene #114454), pCMV-PE1 (Addgene # 132774) and pCMV-PE2 (Addgene # 132775) pCMV-PEmax (Addgene #174820) using HiFi DNA Assembly Master Mix [New England Biolabs (NEB)]. All PCRs were performed using Q5 High-Fidelity DNA Polymerase (NEB). Multi-copy nuclear plasmid pLYW105 for target sites of yeast evolutions is based on pCEV-G1-Ph (Addgene # 46814) and pTBL963_pcDNA3_1 and was assembled by PCR and HiFi DNA Assembly Master Mix containing different epegRNAs and target sites.

Plasmids containing epegRNAs were created by ligation of the annealed spacer, scaffold, and 3’ extension oligos into the BsaI-digested pU6-pegRNA-GG-acceptor (Addgene #13277), pU6-tevopreQ1-GG-acceptor (Addgene #174038) with Golden Gate assembly as previously described^1,3^. To generate intein-split PE plasmids, inserts were ordered as gBlocks from Integrated DNA Technologies (IDT) or amplified from pCMV-PEmax plasmids using PCR. Inserts were cloned into the NotI- and EcoRI-digested pCMV-PEmax backbone using HiFi DNA Assembly Master Mix (NEB). For the cloning of the PiggyBac reporter plasmids for the Adrb1, Dnmt1, PKU and PCSK9 locus, inserts with homology overhangs for cloning were ordered from IDT and cloned into the XbaI- and EcoRI-digested pPB-Zeocin backbone using HiFi DNA Assembly Master Mix (NEB).

To prepare plasmids for AAV production, inserts with homology overhangs were either ordered as gBlocks (IDT) or generated by PCR. Inserts were cloned into XbaI- and NotI-digested AAV backbones using HiFi DNA Assembly Master Mix (NEB).

All plasmids were sequenced by Sanger Sequencing. Oligonucleotides used for cloning all plasmids are listed in Supplementary Table 7. The amino acid sequences of intein-split PEmax p.713/p.714 constructs are listed in Supplementary Table 11.

### Yeast cell culture OD measurements, transformations, and selection

*Saccharomyces cerevisiae* strain GRY333 was a gift from Chang C. Liu with the genotype F102-2, leu∆0, ura3∆0, HIS4 + , his3∆1, flo1∆0 wt-pGKL1/2. All strains were grown at 30 °C in selective complete media or yeast extract peptone (YPD). For selection with Zeocin [100 ug/mL], the pH of the medium was adjusted to 7.5. The plasmids pAR-Ec633 and pLYW105 were transformed into GRY333 as previously described and selected on uracil-depleted plates containing Zeocin^30^. Integration of PE1, PE2 and PEmax on P1 was achieved as previously described with PCR product instead of restriction digested plasmid^6^. After the appearance of colonies, cells were picked and passaged in uracil and leucine-depleted medium containing Zeocin for seven days. The presence and identity of P1 were confirmed by gel electrophoresis of extracted P1 and PCR on the yeast culture. To initiate selections, cultures were diluted 1:1000 in L-histidine-depleted selective complete media. Evolutions were performed in parallel by incubating 200 µL diluted cultures in 96 well plates at 30 °C without shaking. The successful growth of emerging cells was optically identified in the respective wells. Cultures from individual wells were normalized to an OD600 of 0.5 and pooled for PCR amplification of the P1 with the Phire Plant direct PCR master mix (Thermo Fisher). The resulting PCR products were gel purified and transformed into fresh host cells for subsequent rounds of directed evolution, Oxford Nanopore sequencing, or directly subcloned into P2A-GFP fusion plasmid for further characterizations.

### Characterizations of selection plasmid in yeast cell culture

Selection cassettes pLYW105 with and without essential edit were characterized in L-histidine depleted media containing Zeocin by continuous culturing and measurement of absorbance at 600 nm in flat 96 well plates (Greiner) in a Tecan Infinite 200Pro at 30 °C (Supplementary Fig. 1).

### Expression and purification of tethered and untethered prime editor constructs

The tethered and untethered Prime Editor constructs were expressed in *Escherichia coli* Rosetta 2 (DE3) for 18 h at 18 °C as fusion proteins with an N-terminal His6–MBP–TEV tag. Bacterial pellets were resuspended and lysed in 20 mM HEPES-KOH pH 7.8, 500 mM NaCl, 10 mM imidazole, and 5% (v/v) glycerol supplemented with protease inhibitors. Cell lysates were clarified by ultracentrifugation at 19,000 g for 25 minutes at 4 °C, loaded on a 10 mL Ni-NTA Superflow column (QIAGEN) and washed with 5–7 column volumes of 20 mM HEPES-KOH pH 7.5, 500 mM NaCl, 15 mM imidazole. The tagged proteins were eluted with 7–10 column volumes of 20 mM HEPES-KOH pH 7.5, 250 mM NaCl, 250 mM imidazole. The proteins were then loaded on an equilibrated HiTrap Heparin HP column (GE Healthcare). The column was washed with 5 column volumes of 20 mM HEPES-KOH pH 7.5, 250 mM NaCl, 1 mM DTT, and the proteins were eluted with 30 column volumes of 20 mM HEPES-KOH pH 7.5, 1 M NaCl, 1 mM DTT, in a 0–100% gradient. The NaCl concentration was adjusted to 400–500 mM NaCl by dilution and His6–MBP tag was removed by TEV protease cleavage at 4 °C. The proteins were then concentrated and further purified by gel filtration, eluting in 20 mM HEPES pH 7.5, 500 mM NaCl, and 1 mM DTT. pLYW320 (PEmax), pLYW320_Y18 (PE_Y18), pLYW320H (PE_Y17), and pLYW321(PEmax nCas9) were purified using a Superdex 200 16/600 column (GE Healthcare). pLYW321H (PE_Y17 nCas9) and pLYW321_Y18 (PE_Y18 nCas9) were purified using a Superdex 200 26/600 column (GE Healthcare). pLYW322 (PEmax M-MLV RT) and pLYW322H (PE_Y17 / PE_Y18 M-MLV RT) were loaded on a Superdex 75 16/600 gel filtration column (GE Healthcare). Pure fractions were concentrated to 1.4–23.3 mg/mL, analyzed by SDS-PAGE (Supplementary Fig. 5), and flash frozen in liquid nitrogen for storage at −80 °C.

### Screening and validation of enzymatic activities

PE variants were subcloned from normalized yeast cultures from the last round of directed evolutions into the vector pLYW315. 2000 ng of the constructs were transfected into HEK293T in triplicates in 48 well plates. Cells were lysed as previously described with lysis buffer (20 mM Hepes pH7.5, 100 mM KCl, 5 mM MgCl2, 5%(v/v) glycerol, 1 mM DTT, 0.1% (v/v) Triton X-100 and protease inhibitor as previously reported^9^. In vitro transcription was performed on PCR amplified and gel purified pegYW060 with the HiScribe T7 High Yield RNA synthesis kit (NEB). RNA integrity was identified by gel electrophoresis. For lysates, GFP expression levels were normalized with lysis buffer to an equivalent of 150 nM fluorescein as previously described. RNP complexes were formed at room temperature with 1 µL of 2.5 µM pegRNA and 1.25 µL of the normalized lysates respectively. For the purified proteins, 2.5 µM of each construct in 10 mM HEPES, 150 mM NaCl, pH 7.5 was incubated with 2.5 µM pegRNA. The dsDNA substrate was annealed in nuclease-free water by heating to 98 °C and cooling down by > 5 °C per minute. Per reaction 0.5 µL of the annealed dsDNA substrate at varying concentrations was mixed with 0.5 µL of cleavage buffer (100 mM HEPES, pH 7.5, 1.5 M NaCl and 50 mM MgCl_2_) and 1.75 µL of nuclease-free water. The reactions were carried out for 1 h at 37 °C by combining the 2.25 µL of the RNP solution and the 2.75 µL of the substrate solution. The reactions were stopped by the addition of 5 µL 1% Proteinase K (20 mg/mL) followed by incubation at 60 °C for 1 h and 10 minutes at 95 °C.

### Quantification by qPCR

Reverse transcribed cDNA was quantified by qPCR after the enzymatic reactions were stopped. qPCR was performed using the FIREPol qPCR Master Mix (Solis BioDyne) and analyzed using a Lightcycler 480 system (Roche). Quantifications of virtual products were performed with the formula: 10^((Ct-14.87/-3.48)^ *1'000'000.

### Mammalian cell culture, transfection, nucleofections and genomic DNA preparation

HEK293T [American Type Culture Collection (ATCC) CRL-3216] cells were maintained in DMEM plus GlutaMAX (Thermo Fisher Scientific), supplemented with 10% (v/v) fetal bovine serum (FBS) (DMEM + +) (Sigma Aldrich) and 1% penicillin/strepromycin (Thermo Fisher Scientific) at 37 °C and 5% CO_2_. K562 cells (ATCC CCL-243) were maintained in RPMI + + (RPMI 1640 Medium with GlutaMAX Supplement (Thermo Fisher Scientific), supplemented with 10% (v/v) FBS and 1% penicillin/streptomycin. Cells were passaged every 3 to 4 days at a confluency below 90%.

Cells were seeded in 96-well and 48-well cell culture plates at 70% confluency (Greiner) six hours prior to lipofection. Cells were transfected as previously reported and harvested by lysis at respective time points post-transfection with direct lysis buffer: 10 uL of 4x lysis buffer (10 mM Tris-HCl pH 8, 2% Triton^TM^ X-100, 1 mM EDTA, 1% Proteinase K [20 mg/mL])^1^. When intein-split PEs were transfected, 300 ng of each PE half was used.

Nucleofections of HEK293T cells were performed using the Neon^TM^ transfection system using 10 µL tips. Cells were harvested and washed 3 x with phosphate-buffered saline (PBS) prior to counting. Cells were repeatedly spun down and resuspended in R buffer to a concentration of ~ 2*10^5^ cells per 5 µL. Reactions were prepared in PBS by the respective addition of mRNA, proteins, or RNPs consisting of synthetic pegRNA and proteins. For nucleofections 0.125 pmol mRNA and 5 pmol of RNP 1:1 protein:pegRNA molar ratio was used. RNPs were assembled for 10 minutes in PBS at 37 °C. Synthetic pegRNA was ordered at Axolabs (Supplementary Table 11). For mRNA one pulse of 1400 mV and 20 mS pulse width was used and for proteins and RNP one pulse of 1700 mV, 20 mS was applied. After nucleofection, cells were cultured in 200 uL of DMEM + + for 48 hours prior to harvesting.

Genomic DNA from cortices was isolated by phenol/chloroform extraction. First, tissue samples were incubated overnight in lysis buffer (50 mM Tris-HCl pH 8.0, 100 mM EDTA, 100 mM sodium chloride, and 1% SDS; Thermo Fisher Scientific) at 55 °C and 300 rpm. Subsequently, phenol/chloroform/isoamyl alcohol (25:24:1, Thermo Fisher Scientific) was added and samples were centrifuged (5 min, 21,000 *g*). The upper phase was transferred to a clean tube and DNA was precipitated using 100% ethanol (Sigma-Aldrich). Samples were centrifuged (5 min, 21,000 *g*) and pellets were washed using cold 70% ethanol (−20 °C).

### Generation of reporter cell lines

To generate site 1 (Adrb1), site 6 (PKU) and self-targeting site 5 (PCSK9) reporter cell lines with the PiggyBac transposon, HEK293T cells were seeded into a 48-well cell culture plate (Greiner) and transfected at 70% confluency with 225 ng of the PiggyBac-transposon and 25 ng of the transposase using Lipofectamine 2000 (Thermo Fisher Scientific) according to the manufacturer’s instructions. Three days after transfection, cells were enriched for 10 days using Zeocin selection [750 μg/ml]. rSTOP-R2 was generated as preciously described^14^.

### Self-targeting libraries

The custom oligonucleotide pool containing pegRNAs and corresponding target sequence was ordered from Twist Bioscience and cloned into the Lenti_gRNA-Puro plasmid (Addgene #84752) as previously described^12,31^. Cell pools were harvested 120 hours after plasmid transfection without antibiotic selection prior to analysis by deep sequencing.

### AAV production

AAV9 serotype PHP.eB were produced by the Viral Vector Facility of the Neuroscience Center Zurich. Briefly, AAV vectors were ultracentrifuged and diafiltered.To generate Pseudotyped AAV9 vectors, packaging, capsid, and helper plasmids (Addgene No. 112865 and 112867) were co-transfected and incubated for five days until harvest. The vectors were then precipitated using PEG and NaCl and subjected to gradient centrifugation with OptiPrep (Sigma-Aldrich) for further purification, following the previously described method. Subsequently, the concentrated vectors were obtained using Vivaspin® 20 centrifugal concentrators (VWR). Physical titers (vector genomes per milliliter, vg/mL) were determined using a Qubit 3.0 fluorometer (Thermo Fisher Scientific) as previously done^32^. The identity of the packaged genomes of each AAV vector was confirmed by PCR. AAV9 viruses were stored at −80 °C until they were used. If required, they were diluted using phosphate-buffered saline (PBS) from Thermo Fisher Scientific.

### mRNA synthesis and LNP production

An mRNA production plasmid was used to subclone the coding sequences of PEmax, PE_Y17 and PE_Y18, employing HiFi DNA Assembly Master Mix from NEB. Modified nucleoside-containing mRNA was generated using N1mΨ-5′-triphosphate (TriLink) instead of UTP. Co-transcriptional addition of the trinucleotide cap1 analog, CleanCap (TriLink), was used to cap the in vitro transcribed mRNAs. The mRNA was purified by cellulose (Sigma-Aldrich) and analyzed using agarose gel electrophoresis prior to storage at −20 °C. mRNA-LNPs were synthesized by means of nanoprecipitation as reported previously. Briefly, lipids dissolved in ethanol are rapidly mixed with m1Psi modified mRNA dissolved in an aqueous buffer of low pH using a special microfluidic device. mRNA-LNPs were similar in composition to those of the BioNTech vaccine and contained the ionizable lipid ALC-0315 (proprietary to Acuitas Therapeutics), DSPC, Cholesterol and DMG-PEG2000 at 46.3:9.4:42.7:1.6 mol:mol ratio, encapsulated at an RNA to total lipid ratio of ~0.04 (wt/wt). The LNP formulations were dialyzed overnight at 4 °C against 1× PBS, 0.2-μm sterile filtered and stored at 4 °C. Encapsulation efficiencies of mRNA were measured by the Quant-iT Ribogreen Assay (Life Technologies) and LNP sizes were determined with a Malvern Zetasizer (Malvern Panalytical). Polydispersity indexes (PDIs) were determined to be around 0.14 with a Z-average of around 130 nm.

### Animal studies

Animal experiments were performed in accordance with protocols approved by the Kantonales Veterinäramt Zürich and in compliance with all relevant ethical regulations. C57BL/6 J mice were housed in a pathogen-free animal facility at the Institute of Pharmacology and Toxicology of the University of Zurich. Mice were kept in a temperature- and humidity-controlled room on a 12-hour light-dark cycle. Mice were fed a standard laboratory chow (Kliba Nafag no. 3437 with 18.5% crude protein). To target Adrb1 in the brain, newborn mice (P1) received 5.0 × 10^10^ vg per animal and construct via intracerebroventricular injection. For Dnmt1, newborn mice (P1) received 1.7 × 10^10^ vg per animal and construct (Supplementary Fig. 8i-iv) via temporal vein injections. Adult mice were injected with 5 × 10^10^ vg per animal of scAAV9 (Supplementary Fig. 8v). After four weeks animals were dosed with 2 mg/kg (LNP) via the tail vein.

### Brain isolation

Mice were euthanized with CO_2,_ followed by decapitation. The skull was removed with scissors and tweezers without inflicting damage to the underlying tissue. The brain was removed using a spatula. The cortex was identified based on the mouse brain atlas and separated from the remaining brain regions for genomic DNA isolation^33^.

### Isolation of hepatocytes

The process of isolating primary hepatocytes involved a two-step perfusion method. Initially, the liver was subjected to pre-perfusion with Hanks’ buffer, supplemented with EDTA and Hepes, by inserting the cannula through the superior vena cava and cutting the portal vein. This was followed by a low-flow perfusion with digestion buffer containing freshly added Liberase, which lasted for approximately 10 minutes. The digestion was halted using isolation buffer, and the cells were gently scraped away from the matrix using a cell scraper. Subsequently, the cell suspension was filtered through a 100-μm filter from Corning, and the hepatocytes were purified using two low-speed centrifugation steps that lasted for 2 minutes at 50 g.

### Isolation genomic DNA from mouse samples and short read deep sequencing

Genomic DNA from mouse samples was isolated by direct lysis (cells, tissues and isolated hepatocytes) or phenol/chloroform extraction (brain tissue). Specific primers were used to generate targeted amplicons for deep sequencing. Input genomic DNA was first amplified in 10 μL reactions for 30 cycles using NEBNext High-Fidelity 2×PCR Master Mix (NEB). Amplicons were then purified using AMPure XP beads (Beckman Coulter) and subsequently amplified for eight cycles using primers with sequencing adapters. Approximately equal amounts of PCR products were pooled, gel purified, and quantified using a Qubit 3.0 fluorometer and the dsDNA HS Assay Kit (Thermo Fisher Scientific). Paired-end sequencing of purified libraries was performed on an Illumina Miseq. Primers for deep sequencing are listed in Supplementary Table 8.

### HTS data analysis

Sequencing reads were demultiplexed with the Miseq Reporter (Illumina). Next, amplicons were aligned to the respective reference sequence using CRISPResso2 (Clement 2019). Prime editing efficiencies were calculated as percentage of (number of reads containing only the desired edit)/(number of total aligned reads). Indel rates were calculated as percentage of (number of indel-containing reads)/(total aligned reads). Reference amplicons are listed in Supplementary Table 6. Analysis for self-targeting libraries was performed with a custom Python script which will be deposited on.

### Oxford nanopore sequencing

Oxford Nanopore Sequencing was adapted from an established protocol^8^. PE2 variants were extracted by direct PCR amplification from cultured yeast cells from a single 96 well with primers including the respective unique molecular identifiers (UMIs) (Supplementary Table 8). 25 cycles were performed prior to gel extraction and HiFi DNA Assembly into the pUC19 (New England Biolabs) which was previously digested (KpnI-HF, SpHI-HF). For each PCR amplification, approximately 1000 colonies were inoculated prior to plasmid purification. 100 ng of the plasmid pool was PCR amplified with 15 cycles NEBNext High Fidelity 2x PCR Master Mix and corresponding primers with binding regions outside of the UMIs and experiment specific barcodes. Thereafter, Oxford Nanopore sequencing was performed on the as previously described. Consensus reads were created by a previously described Python script^8^ and mutations were counted and identified with a custom Python script that will be deposited on GitHub prior to publication.

### Statistical analysis

All statistical analyzes were performed using GraphPad Prism 9.5.0 for macOS. If not stated differently data represents biological replicates and are depicted as means±s.d. Statistical analyzes are indicated in the respective figure legends. Same applies for the sample sizes and the statistical tests performed respectively. For all analyzes, *p* < 0.05 was considered statistically significant.

### Reporting summary

Further information on research design is available in the Nature Portfolio Reporting Summary linked to this article.

### Supplementary information

Supplementary Information

Peer Review File

Reporting Summary

### Source data

Source Data

## Data Availability

The data generated in this study have been deposited in the National Center for Biotechnology Information Sequence Read Archive (SRA) under accession code PRJNA1034816 [https://www.ncbi.nlm.nih.gov/bioproject/PRJNA1034816]. Source data are provided with this paper.

## Code availability

The custom Python script developed for amino acid identification and counting after clustering the sequences from Oxford Nanopore sequencing is available on GitHub (https://github.com/GeneEditor/PyMutCount).
