# Supplementary material for: Enhancing prime editor activity by directed protein evolution in yeast
Source: Nat Commun. 2024 Mar 7;15:2092. doi: 10.1038/s41467-024-46107-z (PMC10920827; doi:10.1038/s41467-024-46107-z)
Supplement: Supplementary file 1 — Supplementary Information [file 41467_2024_46107_MOESM1_ESM.pdf]

# Enhancing prime editor activity by directed protein evolution in yeast

**Authors:** Yanik Weber[1], Desirée Böck[1], Anastasia Ivascu[2], Nicolas Mathis[1], Tanja Rothgangl[1], Eleonora I. Ioannidi[1], Alex C. Blaudt[2], Lisa Tidecks[1], Máté Vadovics[3], Hiromi Muramatsu[3], Andreas Reichmuth[1], Kim F. Marquart[1,4], Lucas Kissling[1], Norbert Pardi[3], Martin Jinek[2], Gerald Schwank[1]

**Affiliations:**

[1]Institute of Pharmacology and Toxicology, University of Zurich, Zurich, Switzerland
[2]Department of Biochemistry, University of Zurich, Zurich, Switzerland
[3]Department of Microbiology, Perelman School of Medicine, University of Pennsylvania, Philadelphia, PA, USA
[4]Institute of Molecular Health Sciences, ETH Zurich, Zurich, Switzerland

**Supplementary Information**

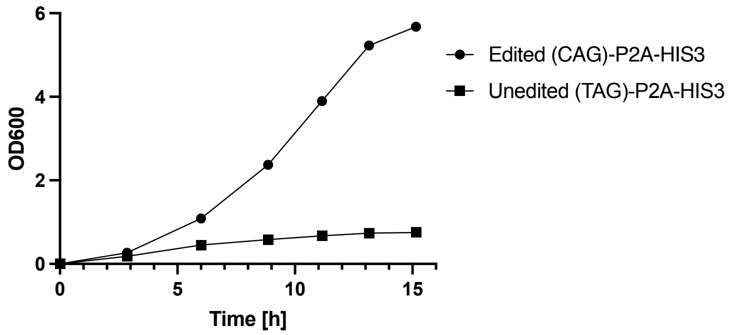


**Supplementary Figure 1: Characterization of the yeast cell growth in the OrthoRep PE selection approach.** The yeast strain GRY333 containing the auxotrophic marker gene HIS3 on a multicopy nuclear plasmid with- and without a stop codon is incubated in L-histidine depleted media. Growth, measured via optical density at 600 nm (OD600) is drastically reduced in yeast cells containing Imidazoleglycerol-phosphate dehydratase (HIS3) copy with the "unedited" stop codon. Each data point corresponds to a measurement from a single experiment (n = 1).

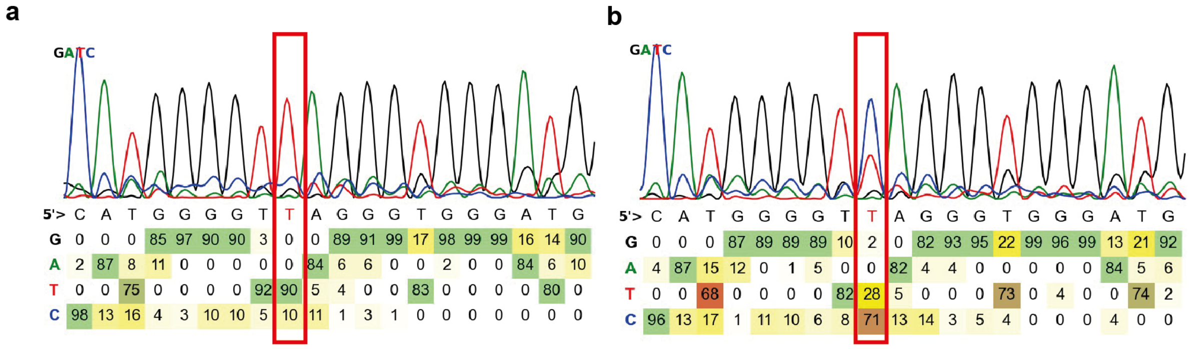


**Supplementary Figure 2: Prime editing of the HIS locus by PE1 during OrthoRep selection.** (**a**) Sanger sequencing of the targeted region (T-to-C transversion) in the auxotrophic marker gene HIS3. (**b**) Prime editing rates after a 5-day incubation period where yeast cells are grown in selective conditions. The chromatograms were analyzed with the base editing analysis tool (BEAT)[1].

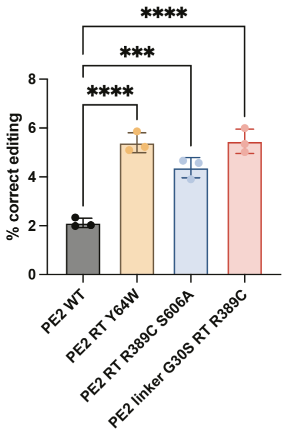


**Supplementary Figure 3: Editing rates with PE2 variants in the HEK293T rSTOP-R2 cell line after a single round of evolution.** Editing rates of PE2 variants isolated after a single round of evolution tested on rSTOP-R2 in HEK293T cells[2]; ****P < 0.0001, ***P = 0.0002 and ****P < 0.0001 (left to right). Data are displayed as means±s.d. of three independent experiments (n=3) and was analyzed using a one-way Anova using Tukey's multiple comparison.

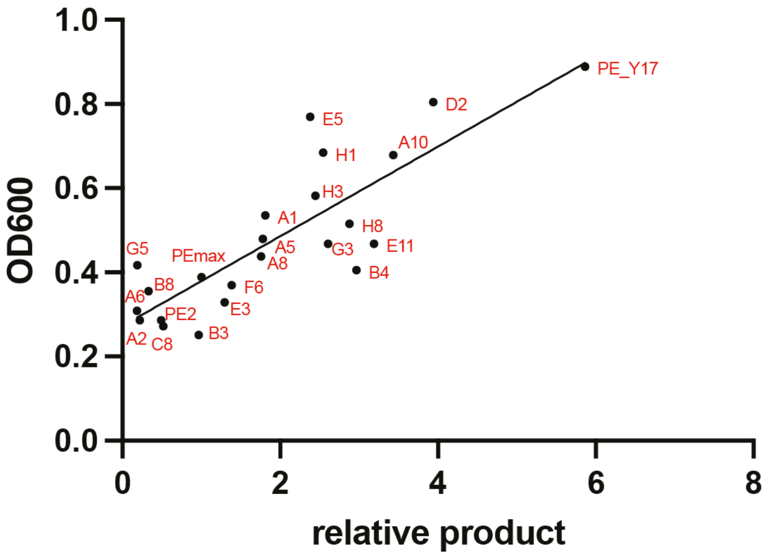


**Supplementary Figure 4: Correlation of yeast growth (under selection) and DNA flap generation in PEKIN of different PE variants.** Measured optical density at 600 nm (OD600) at the final time point (22.6 h) of PEmax variants (Fig 1c) and respective PEKIN activities (Fig 1f) were plotted against each other. The yeast growth in selective conditions correlated with the relative product formation in PEKIN, $P < 0.0001$ and $R^2 = 0.67$. Each data point corresponds to a single experiment (n = 1).

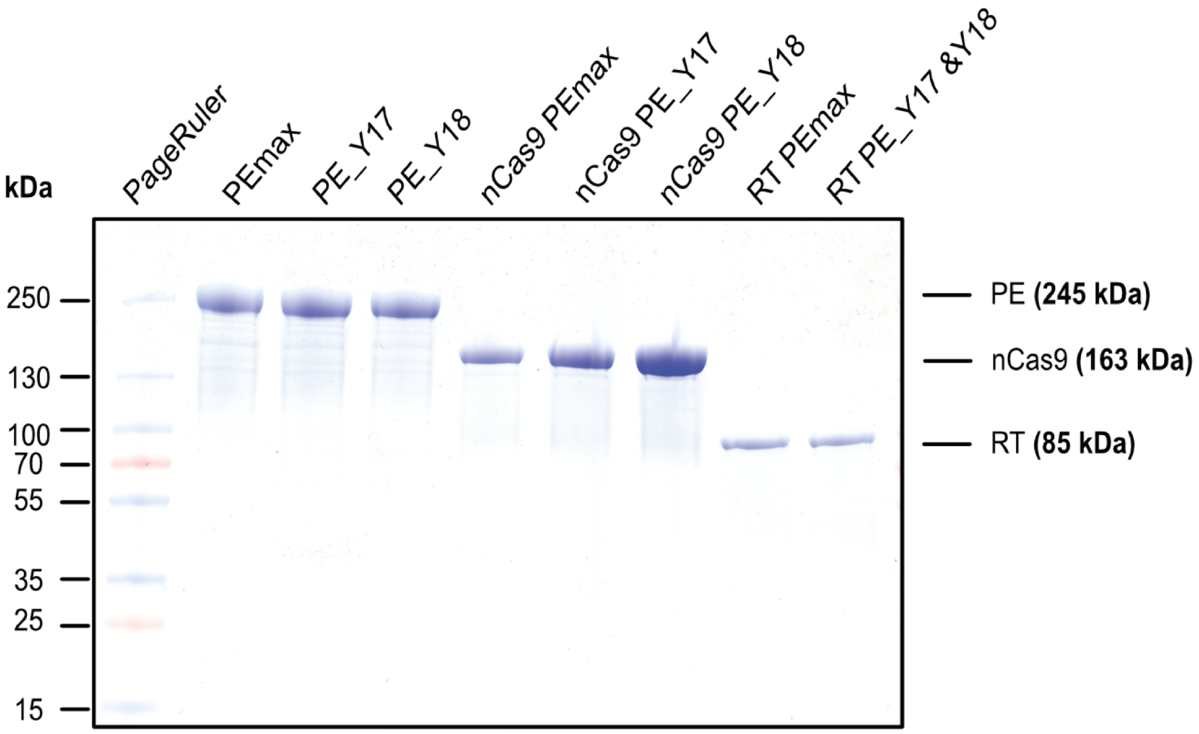


**Supplementary Figure 5: SDS-PAGE analysis of purified Prime Editor proteins.** Gel confirming size, purity, and integrity of isolated proteins. Proteins were expressed in *Escherichia coli* from following plasmids (in order): pLYW320, pLYW320-H, pLYW320-Y18, pLYW321, pLYW321-H, pLYW321-Y18, pLYW322, pLYW322-H).

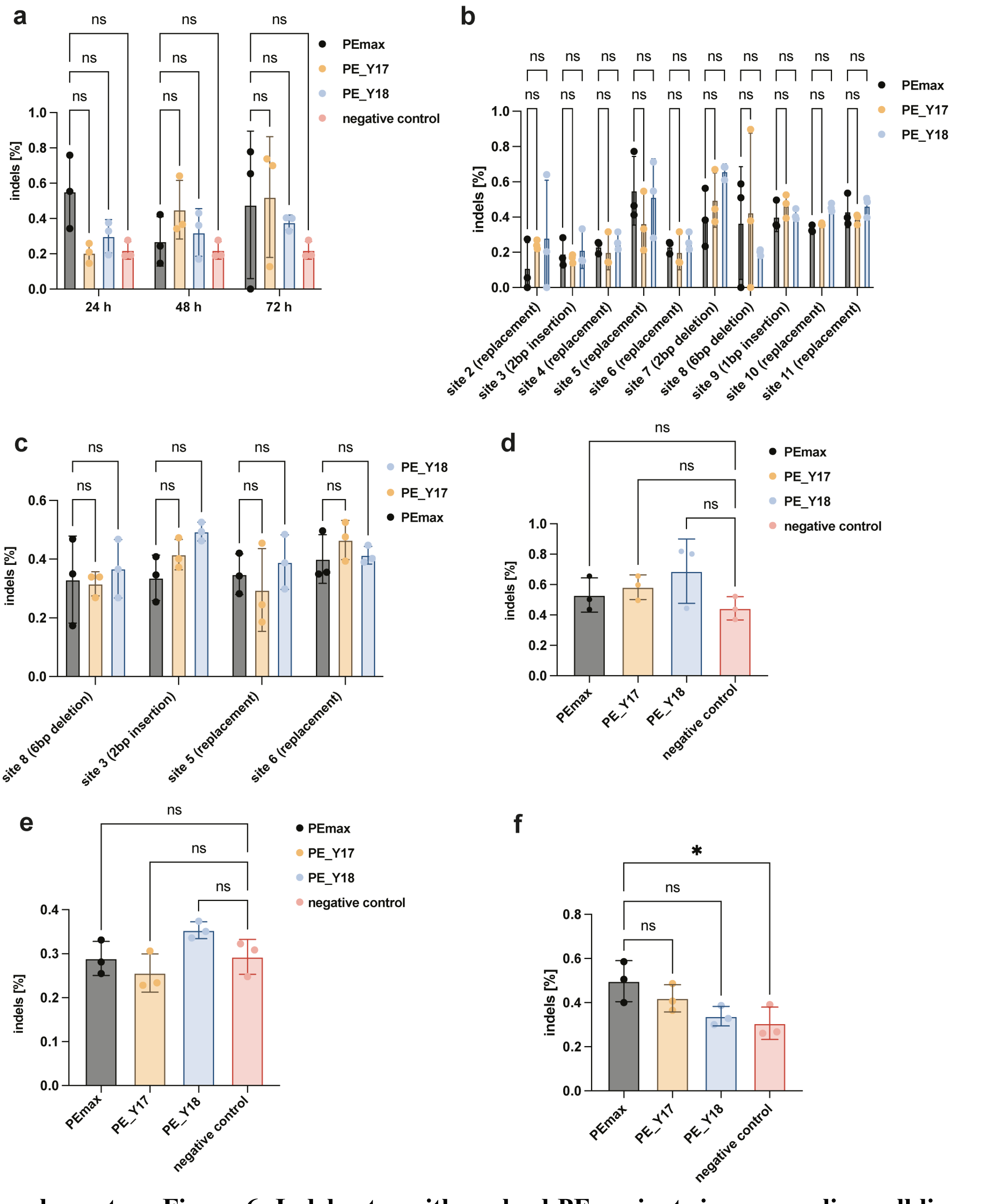


**Supplementary Figure 6: Indel rates with evolved PE variants in mammalian cell lines.** (**a**) Indels at site 1 in HEK293T cells after PE plasmid delivery; not significant (ns) P = 0.5236, ns P = 0.873, ns P = 0.5832, ns P = 0.9865, ns P > 0.9999, ns P > 0.9999, ns P > 0.9999, ns P > 0.9999 and ns P = 0.8588 (left to right). (**b**) Indels in HEK293T cells after PE plasmid delivery; not significant (ns) P = 0.5276, ns P = 0.3463, ns P = 0.9726, ns P = 0.9845, ns P = 0.9643, ns P = 0.9678, ns P = 0.2892, ns P = 0.9532, ns P = 0.9643, ns P = 0.9678, ns P = 0.6775, ns P = 0.0835, ns P = 0.8781, ns P = 0.3643, ns P = 0.8572, ns P = 0.9939, ns P = 0.9721, ns P = 0.5794, ns P = 0.9354 and ns P = 0.9651 (left to right). (**c**) Indels in K562 cells after PE plasmid delivery; not significant (ns) P = 0.9766, ns P = 0.8597, ns P = 0.5067, ns P = 0.0836, ns P = 0.7292, ns P = 0.8273, ns P = 0.633 and ns P = 0.9816 (left to right). (**d**) Indels at site 12 in HEK293T cells expressing the respective pegRNA and nucleofected with mRNA

encoding PEmax, PE_Y17 or PE_Y18; not significant (ns) P = 0.8268, ns P = 0.5619 and ns P = 0.1797. (**e**) Indels at site 12 in HEK293T cells expressing the respective pegRNA and nucleofected with PEmax-, PE_Y17- or PE_Y18 protein; not significant (ns) P = 0.9992, ns P = 0.6243 and ns P = 0.2544. (**f**) Indels at site 4 in HEK293T cells nucleofected with a PEmax-, PE_Y17- or PE_Y18 RNP; not significant (ns) P = 0.2756, ns P = 0.9416 and *P = 0.0422. Data are displayed as means±s.d. of three independent experiments (n = 3) and were analyzed using a one-way Anova using Tukey's multiple comparison.

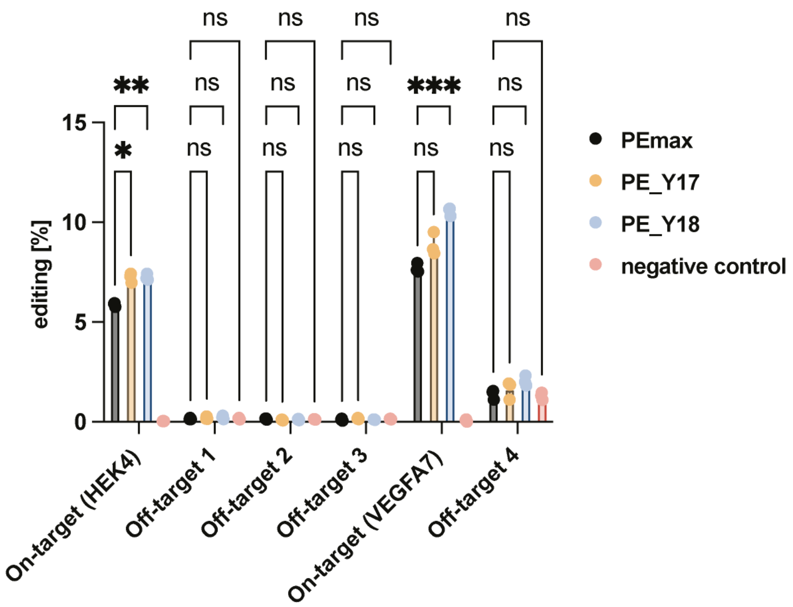


**Supplementary Figure 7: Off-target activity assessed by amplicon sequencing in HEK293T cells.** *P = 0.0103, **P = 0.0018, not significant (ns) P = 0.9995, ns P = 0.9012, ns P = 0.7136, ns P = 0.5955, ns P = 0.4185, ns P = 0.9594, ns P = 0.8218, ns P = 0.6326, ns P = 0.3414, ns P = 0.1478, ***P = 0.0003, ns P = 0.9468, ns P = 0.6678 and ns P = 0.0513 (left to right). Data are displayed as means±s.d. of three independent experiments (n = 3) and were analyzed using a one-way Anova using Tukey's multiple comparison.

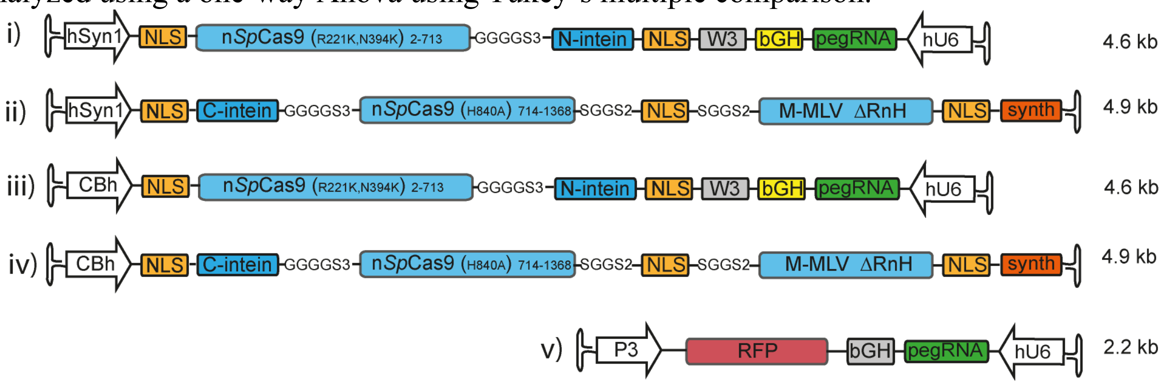


**Supplementary Figure 8: Schematic of the AAV vector designs used in Fig. 4.** Shown are the intein-split designs for the PEs including the nuclear localization signal (NLS) expressed via human synapsin promoter 1 (hSyn1) (i-ii) and the hybrid chicken-beta-actin (CBh) (iii-iv) promoter for PEmax$^{\Delta RnH}$. The designs for PE_Y18$^{\Delta RnH}$ are identical but include the A259D mutation in nCas9 and the K445T mutation in the M-MLV RT respectively. scAAV2/9 design for red fluorescent protein (RFP) expression under the p3 promoter and the enhanced prime editing guide RNA (epegRNA) Dnmt1 expression under the human U6 (hU6) promoter. Terminators included either the W3 and bGH element or used a synthetic polyA tail (synth) was used.

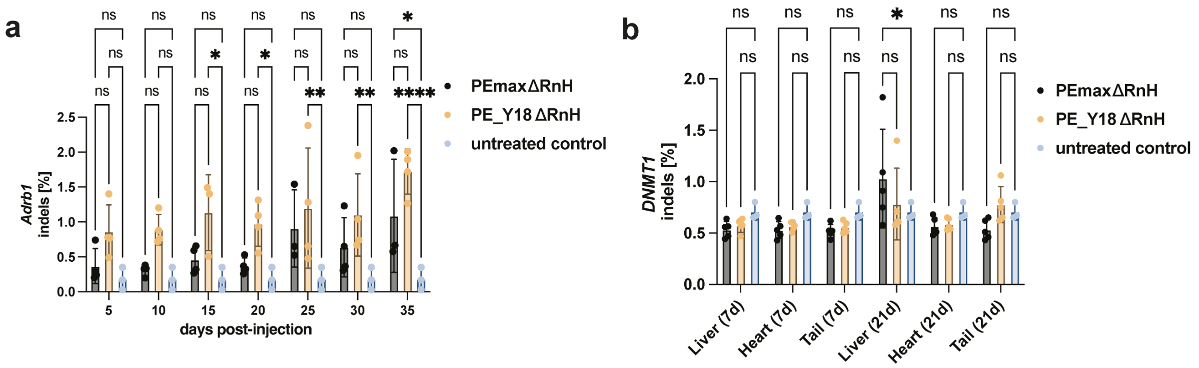


**Supplementary Figure 9: Indel rates with evolved PE variants *in vivo* in mice.** Indel rates were quantified from *in vivo* samples targeting the (**a**) Adrb1 and (**b**) Dnmt1 site, respectively. The number of individual replicates is the following for (**a**) 5–35 days for PEmax$^{\Delta RnH}$: (n= 4),(n = 4), (n = 4), (n = 4), (n = 3), (n = 4), (n = 3) and for PE_Y18$^{\Delta RnH}$: (n = 4), (n = 4), (n = 3), (n = 4), (n = 4), (n = 4), (n = 4); not significant (ns) P = 0.1918, ns P = 0.8121, ns P = 0.0723, ns P = 0.1046, ns P = 0.9048, ns P = 0.0578, ns P = 0.0741, ns P = 0.6305, *P = 0.0128, ns P = 0.0876, ns P = 0.8083, *P = 0.0301, ns P = 0.5961, ns P = 0.073, **P = 0.0039, ns P = 0.2313, ns P = 0.2936, **P = 0.0099, ns P = 0.0979, *P = 0.0187 and ****P < 0.0001 (left to right). The number of individual replicates for (**b**) is: (n = 7) for PEmax$^{\Delta RnH}$ treated mice at day 7, 10 for PE_Y18$^{\Delta RnH}$ treated mice at day 7, and (n = 5) for PEmax and PE_Y18$^{\Delta RnH}$ at day 21. The untreated controls for (**a**) and (**b**) are three mice (n = 3) that were sacrificed at the age of 5 weeks; ns P = 0.3683, ns P = 0.5423, ns P = 0.3529, ns P = 0.5168, ns P = 0.3062, ns P = 0.4616, *P = 0.0345, ns P = 0.8228, ns P = 0.51, ns P = 0.5983, ns P = 0.3651 and ns P = 0.8395. For the treated groups data are displayed as means±s.d. per timepoint and were analyzed using a one-way Anova using Tukey's multiple comparison.

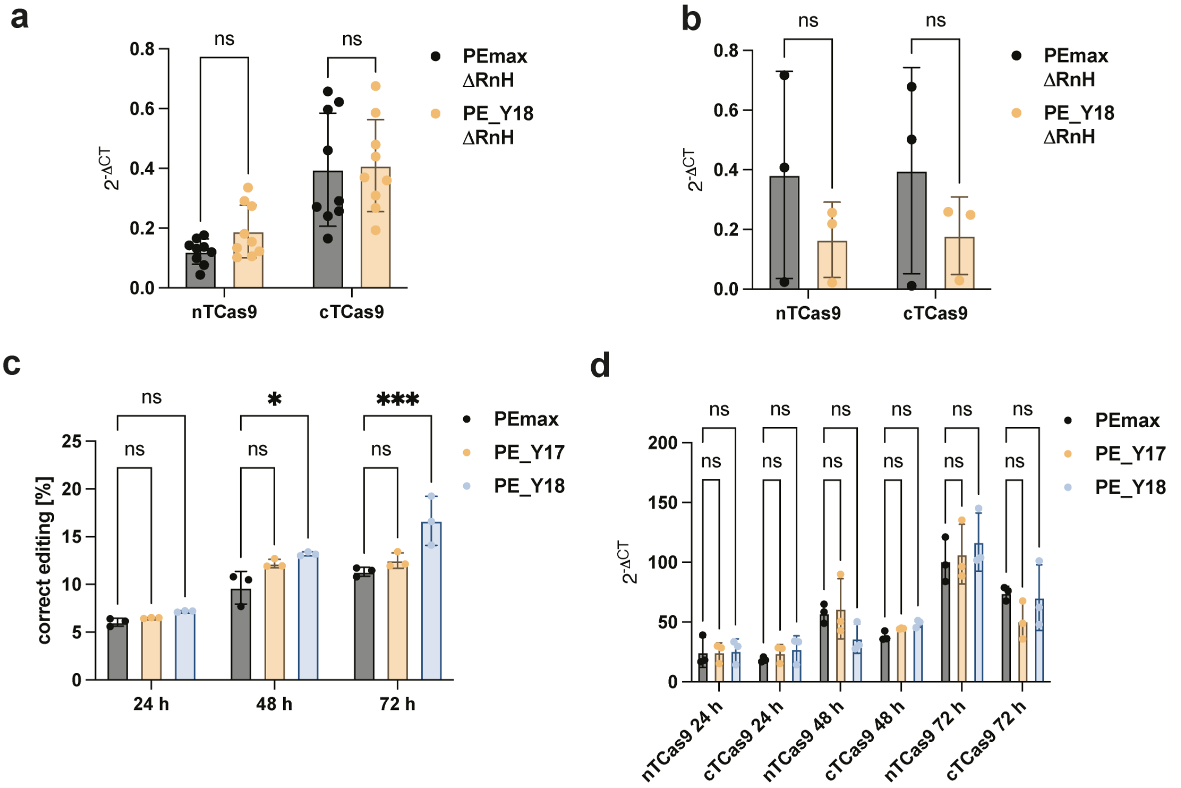

**Supplementary Figure 10: Comparison of expression levels of PE variants.** Expression levels of n-terminal (nTCas9) and c-terminal (cTCas9) AAV constructs were normalized to murine and human GAPDH expression respectively. (**a**) Expression levels of PEmax$^{\Delta RnH}$ and PE_Y18$^{\Delta RnH}$ in the brain of nine randomly selected samples (n = 9); not significant (ns) P = 0.4880 and ns P = 0.9714 (left to right). (**b**) Expression levels of PEmax$^{\Delta RnH}$ and PE_Y18$^{\Delta RnH}$ in the liver of three randomly (n=3) selected samples; not significant (ns) P = 0.6306 and ns P = 0.6275 (left to right). (**c**) Intended editing at site 12 with PEmax, PE_Y17 and PE_Y18 in HEK293T cells 24, 48 and 72 h after transfections in three (n = 3) independent biological replicates; not significant (ns) P = 0.9999, ns P = 0.9318, ns = 0.1737, *P = 0.0204, ns P = 0.9158 and P = 0.0003 (left to right).(**d**)Expression levels of PE variants transfected in panel (**d**) assessed by RT-qPCR of three (n = 3) independent biological replicates; not significant (ns) P > 0.9999, ns P > 0.9999, ns P > 0.9999, ns P > 0.9999, ns P > 0.9999, ns P = 0.9448, ns P > 0.9999, ns P > 0.9999, ns P > 0.9999, ns P = 0.9961, ns P = 0.8847 and ns P > 0.9999 (left to right). Data are displayed as means±s.d. of replicates per timepoint and were analyzed using a one-way Anova using Tukey's multiple comparison.

## Supplementary Tables

**Supplementary Table 1: Mutations generated during PE evolution in OrthoRep and identified through Sanger sequencing.** List of identified mutations from Sanger sequencing of subcloned variants after visible outgrowth of yeast cells in 96 well plates in selective conditions.

| | Amino acid number | aa PE1; PE2; PEMax | PE1 | PE2 | PEmax | Colony number round 1 | Colony number round 4 |
|---|---|---|---|---|---|---|---|
| nCas9 (H840A) | 55 | S | | | T | 1 | |
| | 71 | R | | | S | 11 | 13 |
| | 132 | Y | | | C | 12 | 16 |
| | 197 | E | | G & D | | 14, 15 | |
| | 219 | S | | | A | | 1, 6, 8, 10, 18 |
| | 240 | N | D | | | 35 | |
| | 254 | S | | | A | 10 | |
| | 259 | A | | | D | | 2, 7, 11, 13, 17 |
| | 268 | K | T | | | 34 | |
| | 318 | S | | | N | 9 | 14 |
| | 320 | S | | | R | | 12, 15 |
| | 357 | N | | | T | 9 | |
| | 430 | Y | | H | | 13 | |
| | 469 | S | | | E | 8 | |
| | 478 | F | | V | | 16 | |
| | 694 | M | | | V | 7 | |
| | 800 | P | L | | | 32 | |
| | 876 | V | | | M | 3 | |
| | 1047 | K | R | | | 31 | |
| | 1265 | Y | C | | | 30 | |
| | 1360 | I | | N | | 16 | |
| Linker | 28 | G | D | | | 17 | 10 |
| | 30 | G | | S | | 3 | 7, 13 |
| M-MLV RT | 44 | R | | | H | 2 | 5, 11 |
| | 64 | Y | | C & W | W | 18, 19 | 3, 4, 8 |
| | 147 | T | Y | | | 29 | |
| | 180 | S | | L | | 20 | |
| | 200 | D;N;N | N | | | 28 | |
| | 259 | A | Y | | | 27 | |
| | 330 | T;P;P | P | | | 26 | |
| | 373 | K | | | R | 6 | 3, 15, 17, 18 |
| | 389 | R | | C | C | 3 | 3, 7, 14 |
| | 432 | L | | | M | | 3, 14 |
| | 445 | K | | | T | | 3, 6, 8, 10, 13, 18 |
| | 451 | W | | M & * | | 21, 22 | |
| | 454 | N | K | | | 26 | |
| | 455 | A | P | | | 25 | |
| | 477 | A | | | V | 6 | |
| | 513 | P | S | | | 24 | |
| | 536 | A | | | V | 4 | |
| | 599 | R | A | | | 23 | |
| | 606 | S | | | A | 5 | 7 |

**Supplementary Table 2: Identified mutations in PE variants via long read nanopore sequencing.** PE2 variants were isolated from a single well after selection in OrthoRep and subjected to nanopore sequencing to analyze evolutionary trajectories. Unique molecular identifiers (UMIs) were clustered and used to assess respective variant frequencies.

| UMI counts | nCas9 (PE2) | Linker | M-MLV-RT (PE2) |
|---|---|---|---|
| 478 | | G30S | R389C |
| 381 | | G30S | R389C , A455P |
| 2 | | G30S, G32S | |
| 1 | | G30S | L269G |
| 1 | | G30S | A455P |
| 1 | K999I | G30S | R389C |

## Supplementary Table 3: Overview of parallel OrthoRep evolutions with PEmax.

| Round of evolution | Edit | pegRNA | 2 micron selection plasmid | Number of wells showing outgrowing cells |
|---|---|---|---|---|
| 1 | TAG - CAG | gYW33bB | pLYW105 | 3 |
| 2 | TAG - CAG | gYW33bB | pLYW105 | 40 |
| 3 | TAG - CAG | gYW33bB | pLYW105 | 94 |
| 4 | 501 bp insertion | pegYW074 | pLYW204 | 6 |

## Supplementary Table 4: pegRNA sequence used for evolutions.

| pegRNA name | pegRNA sequence (5' to 3') |
|---|---|
| gYW33bB | AAUGGAGCCACCAUGGGGUUGUUUUAGAGCUAGAAAUAGCAAGUUAAAAUAAGGCUAGUCCGUUAUCAACUUGAAAAAGUGGCACCGAGUCGGUGCUCCCACCCUGACCCCAUGGUGGC |
| pegYW074 | AUUACCGUCUCACUUUGGAUCGAGUGCUCUAUCGCUGUUUCAGAGCUAUGCUGGAAACAGCAUAGCAAGUUGAAAUAAGGCUAGUCCGUUAUCAACUUGAAAAAGUGGCACCGAGUCGGUGCAGAAAGCCCUAGUAAAGCGUAUUACAAAUGAAACCAAGAUUCAGAUUGCGAUCUCUUUAAAGGGUGGUCCCCUAGCGAUAGAGCACUCGAUCUUCCCAGAAAAAGAGGCAGAAGCAGUAGCAGAACAGGCCACACAAUCGCAAGUGAUUAACGUCCACACAGGUAUAGGGUUUCUGGACCAUAUGAUACAUGCUCUGGCCAAGCAUUCCGGCUGGUCGCUAAUCGUUGAGUGCAUUGGUGACUUACACAUAGACGACCAUCACACCACUGAGGACUGCGGGAUUGCUCUCGGUCAAGCUUUUAAAGAGGCCCUAGGGGCCGUGCGUGGAGUAAAAAGGUUUGGAUCAGGAUUUGCGCCUUUGGAUGAGGCACUUUCCAGAGCGGUGGUUGAUCUUUCGAACAGGCCGUACGCAGUUGUCGAACUUGGUUUGCAAAGGGAGAAAGUAGGUGAUCUCUCUUGCGAGAUGAUCCCGCAUUUUCUUGAAAGCUUUGCAGAGGCUAGCAGAAUUACCCUCCACGUUGAUUGUCUGCGAGGCAAGAAUGUUUUAGAGACGGUAUU |

## Supplementary Table 5: Overview of the number of wells showing visible yeast cell growth with PEmax using different types of edits.

| Round of evolution | Edit | pegRNA | 2 micron selection plasmid | Number of wells showing outgrowing cells |
|---|---|---|---|---|
| 1 | TAG - CAG | gYW33bB | pLYW105 | 3 |
| 2 | 501 bp insertion | pegYW074 | pLYW204 | 0 |

## Supplementary Table 6: Nucleotide sequences of HTS amplicons used for alignments.

| name | oligo sequence (5' to 3') |
|---|---|
| Site 1 Adrb1 | CCAGCATTGAGACCCTGTGTGTCATCGCCCTGGACCGCTACCTCGCCATCACGTCGCCCTTTCGCTACCAGAGTTTGCTGACGCGCGCGCGAGCGCGGGCCCTCGTGTGCACAGTGTGGGCCATCTCGGCGTTGGTGTCCTTCCTGCCCATCCTCATG |
| Site 2 INTER3 | CGTCACTTACGGTACACATTCCAAAGGTGGCTCTTGAGAGGACCTCCTCGTGGCAAGCAAGCTCGGATAATTTCAGGACTTTTCAGTCAGTTTCTGTTCAGCTCAGATTATATCAAATTTGAAAGAAGGTGAAACTAACCACGTTAGTGTCATTTCCACTTCATGGGAGATATGTGAACCCCATTATGACAATCTCCTTTATGTTTCATCTAGAAATTT |
| Site 3 & 6 *FOXO3* | ATTTATTCATGTATTTGTGATAAATGCCTAGGAGTAGGACTGCTGGGTGGTAAGTGCAGTGTATGTTTAACTTTATAAGAAACTGCCAAACTGTTTTCCAGAGTGAATGTACTGTTT |
| Site 4 PKU | TCCGTCCTGTTGCTGGCTTACTGTCGTCTCGAGATTTCTTGGGTGGCCTGGCCTTCCGaGTCTcCCACTGCACACAGTACATTAGGCATGGATCTAAGCCCATGTACACACCTGAACCGTAAGTATCATTCTTCAGCTACCCCTGCCAACCACAATGGATGCTCA |
| Site 5 NDEL1 | TTCTATGGGCCTGATGGACTAGGCTTCATCCTGGGACTTTTCATTGCAGCCCTAGGTTGGAGCCCCTATTTCCTGCATCCCATGCTGTCCAGGTCATGGTTTTCTCCCTTCTTTTGTACTTGTCTCAGGA |
| Site 7 & 9 EXO1 | CGTCACTTACGGTACACATTCCAAAGGTGGCTCTTGAGAGGACCTCCTCGTGGCAAGCAAGCTCGACTTATCAGGAAGAGCATTCTGGGCATTTGAGTGCATCAGGAGAGGATGCCCTTCCCATAGCCACTGGCCA |
| Site 10 HEK4 | CGCGGCGCCCCGGTGGCACTGCGGCTGGAGGTGGGGGTTAAAGCGGAGACTCTGGTGCTGTGTGACTACAGTGGGGGCCCTGCCCTCTCTGAGC |

| | |
|---|---|
| Site 11 VEGFA7 | TGCAGACAAACGGAACTCAACCATTAAGCAAAACATGGGAACTCAGTTTATATGAGTTACAACGAACACCTCAGGTAATGACTAAGATGACTGCCAAGGGG |
| Site 12 PCSK9 | CCACGGCACCCACCTGGCAGGTGTGGTCAGCGGCCGGGATGCTGGTGTGGCCAAGGGCACCAGCCTGCACAGCCTGCGTGTGCTCAACTGTCAAGGGAAGGGCACAGTCAGCGGCACCCTCATAGGTGAGTACTCTCTGGGTTCTACC |
| Site 13 FANCF | CGCAGAGAGTCGCCGTCTCCAAGGTGAAAGCGGAAGTAGGGCCTTCGCGCACCTCATGGAATCCCTTCTGCAGCACCTGGATCGCTTTTCCGAGCTT |
| Site rSTOP2 | CACTGTGGTCTCAAGCCTCAGACAGTGGTTCAAAGTTTTTTTCTTCCATTTCAGGTGTCGTGAGTCTAGAGCCACCATGGGACTAGAGTAGGATTGTACCCCTCAGTATGGATCCGGAGGTGGTGGCAGCGGTGGTGGTAGCATAGTGAGCAAGGGCGCGTATTGCA |
| Dnmt1 | GTCTTCCCCCACTCTCTTGCCCTGTGTGGTACATGCTGCTTCCGCTTGCGCCGCCCCCTCCCAATTGGTTTCCGCGCGCGCGAAAAAGCCGGGGTCTCGTTCAGAGCTGTTCTGTCGTCTGCAACCTGCAAGATGCCAGCGCGAACAGCTCCAGCCCGAGTGCCTGCGCTTGCCTCCCCGGCAGGCTCGCTCCCGGACCATGTCCGCAGGCGGTAGGTGCCACGCAGGGTGGGGGTGAGGGGCGGGACCGATGCCGAGGCATATATTGGGGG |
| HEK4 off-target 1 | TCCCTGTCTTTGAATCTTACTTTCCATCCTCCACCTCCTCCGGCCGCAGTGCACCAAGCTGGAAGCAGCACCCTGCAGCCGGGCTGGACCACTCTGGAGAGCAGTGAGCCCAGCAGAGCCCCCCTAGGGATCTTCAGCCCCAGCTATGAGTCTCAGAAGCCATGC |
| HEK4 off-target 2 | AGCCTGTCTTCAGGGCACATGCACGTGCGCAGGGCTCTGCGGCTGGAGGGGGTGGGGTTGCTGTTAGTGACAGGGGCCCCAGCCAGGCAGGTTTCAGGATTGGGGAG |
| HEK4 off-target 3 | CCATGTGCAAACCCCTTCCTGGGCAACCCCGGGTTAGGCCCCCTCCACCTCCAGCCGTGATGCCTTGCCTGTATGTACCAGCCCAGAAAGAACATCCGGGTGCAGGTGATTCCATCCCACAGCA |
| VEGFA7 off-target | CATGAGGAGATTTGCATTTCCAAAAGACCCCCCCACCCCGCCCCCGGCTGCTGTTGCAGAGGACAAGTTGAGGAGGGCAGAGAGGATGTGGAGAGGACAGCAGGGAGTACTGCAGCTGTCCAAGTCA |

## Supplementary Table 7: Oligonucleotides used for cloning.

| oligo name | oligo sequence (5’ to 3’) |
|---|---|
| gYW060_fwd | TAATACGACTCACTATAGGGGCTCACTCATTAGGCACCCCGTTTCAGAGCTATGCTGG |
| gYW060_rev | GATCCAAGCTTTTTAAAACACTCATTAGGCACCCCAGGCTTTACACTACCAGGATGGGCACCACCCCGGTGAACAGCTCCTCGCCCTTGCTCACCATTTAGCACCGACTCGGTGC |
| oYW_001 | CGGATCGACCTGTCTCAGCTGGGAGGTGAC |
| oYW_004 | GTCACCTCCCAGCTGAGACAGGTCGATCCG |
| oYW_1000 | TACTTCCAATCCAATGCAATGAAACGGACAGCCGACGGAAG |
| oYW_1001 | CATTGCATTGGATTGGAAGTACAGGTTTTC |
| oYW_1005 | AAAAAAATTACGCCCCGCCCTGCCA |
| oYW_1006 | AGGGGATCGGGAGATCGATCTCCCGATCCGTCGACGTCAGGTGGCACTTTTCGGGGAAATGTGTGATCGGCACGTAAGAGGTTCCAAC |
| oYW_1007 | CGGGGCGTAATTTTTTTCTGTCAGACCAAGTTTACTC |
| oYW_1011 | CATCACCATCACCATCACTAATAACCAACTC |
| oYW_1012 | GATGGTGATGGTGATGAGAGCCGGATTGGAAGTAGAGGTTCTCGTCCAGCTTCACTCTCTTAGCGGCAGG |
| oYW_1013 | GTGATGGTGATGGTGATGAGAGCCGGATTGGAAGTAGAGGTTCTCGTCACCTCCCAGCTGAGACAGGTCGATCCG |
| oYW_1014 | ACTTCCAATCCAATGCATCCGGCGGAAGCTCTGGTGGCAGCAAG |
| oYW_1017 | CAAGCCACCCCTCTCAGCTTTAGTTAAG |
| oYW_1023 | GCTATCGAAGCCACCGTACTTTTTCGGG |
| oYW_1071 | GGTCTTTTTCACGATATTCACTTGGGG |
| oYW_1076 | ATAGGGAGAGCCGCCACCATGAAACGGACAGCCGACGGAAGCGAGTTCGAG |
| oYW_1084 | CCACAGGGCCAAACTGCACGCG |
| oYW_188 | TACGACGTGGATGCTATCGTGCCCC |
| oYW_189 | GGGGCACGATAGCATCCACGTCGTA |
| oYW_190 | GTGATAAGCTCATAGACATATAAAATGGACAAGAAGTACTCCATTG |
| oYW_191 | CAGCTCGGTGGAGACTCTGGAGGATCTAGCGGA |
| oYW_192 | GCTAGATCCTCCAGAGTCTCCACCGAGCTGAGAG |
| oYW_193 | TGACTCCGGCGAAAAATTAGACTTTCCTCTTCTTCTTGGG |
| oYW_194 | AAGAAGAAGAGGAAAGTCTAATTTTTCGCCGGAGTCAATTA |
| oYW_470 | GCAAGAGCAGAAAGCTGGAAAATCTGATCGCC |
| oYW_472 | TGAAGCTGAAGAGAGAGGACCTGCTGCGG |

| | |
|---|---|
| oYW_550 | GGACCATGGTGCCGGCCCGTGGCCTATC |
| oYW_554 | TGGCTGACCGCCGAGGGCAAGGAGATCAAG |
| oYW_585 | ACTATAATATATGAATTACATTATTAATTTAAAATATACATAGGAAGATCTATAGAAACAAAAAGATTAA TAACTTTCAAATATCAGAAAAATATAGAAACATGTG |
| oYW_673 | GGCTGTCCGTTTCATGGTGGCGGCTCTCCCTATA |
| oYW_885 | GAGATCGCCTGGGATAAGGGCCGGG |
| oYW_911 | GCCTGACCTTTAAAGAGGACATCCAGAAAG |
| oYW_940 | CTTGGACAGATAGGCCACGGGCCGTCTCC |

**Supplementary Table 8: Oligonucleotides used for deep sequencing.**

| oligo name | oligo sequence (5' to 3') |
|---|---|
| HTS-Adrb1-endogenous_fwd | CTTTCCCTACACGACGCTCTTCCGATCTNNNNNNCCAGCATTGAGACCCTGTGT |
| HTS-Adrb1-endogenous_rev | GGAGTTCAGACGTGTGCTCTTCCGATCTNNNNNNCATGAGGATGGGCAGGAAGG |
| HTS-Adrb1-PiggyBac_rev | GGAGTTCAGACGTGTGCTCTTCCGATCTNNNNNNATAGGGCCCTCTAGACGCTT |
| HTS-Dnmt1-invivo_fwd | CTTTCCCTACACGACGCTCTTCCGATCTNNNNNNGTCTTCCCCCACTCTCTTGC |
| HTS-Dnmt1-invivo_rev | GGAGTTCAGACGTGTGCTCTTCCGATCTNNNNNNCCCCCAATATATGCCTCGGC |
| HTS-Adrb1-invivo_fwd | CTTTCCCTACACGACGCTCTTCCGATCTNNNNNNTCGCTACCAGAGTTTGCTGA |
| HTS-Adrb1-invivo_rev | GGAGTTCAGACGTGTGCTCTTCCGATCTNNNNNNAGCACTTGGGGTCGTTGTAG |
| oYW346 (Nanopore UMIs) | CATGATTACGCCAAGCTTGCATGCGATACNNNNNNNNNNNNNNNNNNNNNNNNNG ATACNNNNNNNNNNNNNNNNNNNNNNNNNTGACTCCGGCGAAAAA |
| oYW190(Fwd PE2 Nanopore) | GTGATAAGCTCATAGACATATAAAATGGACAAGAAGTACTCCATTG |
| pNM 653 | CTTTCCCTACACGACGCTCTTCCGATCTGGTGTCATCCGTCTGAAGCA |
| pNM 654 | GGAGTTCAGACGTGTGCTCTTCCGATCTAGGGCTACCTCCTACACACT |
| pNM 664 | CTTTCCCTACACGACGCTCTTCCGATCTAGGCCACACAAACCGTCATA |
| pNM 665 | GGAGTTCAGACGTGTGCTCTTCCGATCTTAAGCTGGTCTCGAGCTCCT |
| pNM 670 | CTTTCCCTACACGACGCTCTTCCGATCTTGTTCAGGACTTTTGGGGATGT |
| pNM 671 | GGAGTTCAGACGTGTGCTCTTCCGATCTACATTGCTGGCAGAAATGCA |
| HTS_PKU_fw | CTTTCCCTACACGACGCTCTTCCGATCTNNNNNNCCGTCCTGTTGCTGGCTTAC |
| HTS_PKU_rev | GGAGTTCAGACGTGTGCTCTTCCGATCTNNNNNNTGAGCATCCATTGTGGTTGG |
| Reporter_NGS_1FW | CTTTCCCTACACGACGCTCTTCCGATCTCACTGTGGTCTCAAGCCTCAGACAGTG G |
| Reporter_NGS_1RV | GGAGTTCAGACGTGTGCTCTTCCGATCTGCAATACGCGCCCTTGCTCACTATGCT A |
| *oYW_1342* | ACACTCTTTCCCTACACGACGCTCTTCCGATCTNNNNGAACCCAGGTAGCCAGAG AC |
| *oYW_1343* | TGGAGTTCAGACGTGTGCTCTTCCGATCTTCCTTTCAACCCGAACGGAG |
| *oYW_1344* | ACACTCTTTCCCTACACGACGCTCTTCCGATCTNNNNGGCATGGCTTCTGAGACT CA |
| *oYW_1345* | TGGAGTTCAGACGTGTGCTCTTCCGATCTGTCTCCCTTGCACTCCCTGTCTTT |
| *oYW_1346* | ACACTCTTTCCCTACACGACGCTCTTCCGATCTNNNNTTTGGCAATGGAGGCATT GG |
| *oYW_1347* | TGGAGTTCAGACGTGTGCTCTTCCGATCTGAAGAGGCTGCCCATGAGAG |
| *oYW_1350* | ACACTCTTTCCCTACACGACGCTCTTCCGATCTNNNNTTTCCACCAGAACTCAGC CC |
| *oYW_1351* | TGGAGTTCAGACGTGTGCTCTTCCGATCTCCTCGGTTCCTCCACAACAC |

**Supplementary Table 9: Oligonucleotides used for synthetic target and qPCR.**

| oligo name | oligo sequence (5' to 3') |
|---|---|
| oYW_741(fwd synthetic target) | AACATACGAGCCGGAAGCATAAAGTGTAAAGCCTGGGGTGCCTAATGAGTGAGCTAACTCACATTA ATTGCGTTGCGCTC |
| oYW_742(rev synthetic target) | GAGCGCAACGCAATTAATGTGAGTTAGCTCACTCATTAGGCACCCCAGGCTTTACACTTTATGCTT CCGGCTCGTATGTT |
| oYW_644(fwd) | GAGCGCAACGCAAT |
| oYW_645(rev) | CCATCCTGGTAGAGCC |

**Supplementary Table 10: Nucleotide sequences of synthetic pegRNA.**

| pegRNA name | oligo sequence (5’ to 3’) |
|---|---|
| PKU Axolabs | gscscsUAAUGUACUGUGUGCAGGUUUCAGAgcuaugcuggaaacagcauagcAAGUUGAAAUAA GGCUAGUCCGUUAUCAacuugaaaaaguggcaccgagucggugcUUCCGAGUCUUUCACUGCACA CAGUACAususas |

N: RNA residues
n: 2'-O-methyl residues
s: phosphorothioate backbone modification

**Supplementary Table 11: Amino acid sequences of intein-split PEmax p.713 and p.714 constructs.**

| Intein-split PEmax p.713: p.NLS/n*Sp*Cas9$^{1\text{-}713}$(R221K,N394K)/linker/N-intein/NLS |
|---|
| MKRTADGSEFESPKKKRKVDKKYSIGLDIGTNSVGWAVITDEYKVPSKKFKVLGNTDRHSIKKNLIG<br>ALLFDSGETAEATRLKRTARRRYTRRKNRICYLQEIFSNEMAKVDDSFFHRLEESFLVEEDKKHERH<br>PIFGNIVDEVAYHEKYPTIYHLRKKLVDSTDKADLRLIYLALAHMIKFRGHFLIEGDLNPDNSDVDKL<br>FIQLVQTYNQLFEENPINASGVDAKAILSARLSKSRKLENLIAQLPGEKKNGLFGNLIALSLGLTPNFK<br>SNFDLAEDAKLQLSKDTYDDDLDNLLAQIGDQYADLFLAAKNLSDAILLSDILRVNTEITKAPLSAS<br>MIKRYDEHHQDLTLLKALVRQQLPEKYKEIFFDQSKNGYAGYIDGGASQEEFYKFIKPILEKMDGTE<br>ELLVKLKREDLLRKQRTFDNGSIPHQIHLGELHAILRRQEDFYPFLKDNREKIEKILTFRIPYYVGPLA<br>RGNSRFAWMTRKSEETITPWNFEEVVDKGASAQSFIERMTNFDKNLPNEKVLPKHSLLYEYFTVYN<br>ELTKVKYVTEGMRKPAFLSGEQKKAIVDLLFKTNRKVTVKQLKEDYFKKIECFDSVEISGVEDRFNA<br>SLGTYHDLLKIIKDKDFLDNEENEDILEDIVLTLTLFEDREMIEERLKTYAHLFDDKVMKQLKRRRYT<br>GWGRLSRKLINGIRDKQSGKTILDFLKSDGFANRNFMQLIHDDSLTFKEDIQKAQVGGGGSGGGGSG<br>GGGSCLSYETEILTVEYGLLPIGKIVEKRIECTVYSVDNNGNIYTQPVAQWHDRGEQEVFEYCLEDGS<br>LIRATKDHKFMTVDGQMLPIDEIFERELDLMRVDNLPNSGGSKRTADGSEFEPKKKRKV* |

| Intein-split PEmax p.714: p.NLS/C-intein/linker/n*Sp*Cas9$^{714\text{-}1368}$(H840A)/linker-NLS-linker/RT-dRnH/NLS |
|---|
| MKRTADGSEFESPKKKRKVIKIATRKYLGKQNVYDIGVERDHNFALKNGFIASNGGGGSGGGGSGG<br>GGSSGQGDSLHEHIANLAGSPAIKKGILQTVKVVDELVKVMGRHKPENIVIEMARENQTTQKGQKN<br>SRERMKRIEEGIKELGSQILKEHPVENTQLQNEKLYLYYLQNGRDMYVDQELDINRLSDYDVDAIVP<br>QSFLKDDSIDNKVLTRSDKNRGKSDNVPSEEVVKKMKNYWRQLLNAKLITQRKFDNLTKAERGGL<br>SELDKAGFIKRQLVETRQITKHVAQILDSRMNTKYDENDKLIREVKVITLKSKLVSDFRKDFQFYKV<br>REINNYHHAHDAYLNAVVGTALIKKYPKLESEFVYGDYKVYDVRKMIAKSEQEIGKATAKYFFYSN<br>IMNFFKTEITLANGEIRKRPLIETNGETGEIVWDKGRDFATVRKVLSMPQVNIVKKTEVQTGGFSKES<br>ILPKRNSDKLIARKKDWDPKKYGGFDSPTVAYSVLVVAKVEKGKSKKLKSVKELLGITIMERSSFEK<br>NPIDFLEAKGYKEVKKDLIIKLPKYSLFELENGRKRMLASAGELQKGNELALPSKYVNFLYLASHYE<br>KLKGSPEDNEQKQLFVEQHKHYLDEIIEQISEFSKRVILADANLDKVLSAYNKHRDKPIREQAENIIHL<br>FTLTNLGAPAAFKYFDTTIDRKRYTSTKEVLDATLIHQSITGLYETRIDLSQLGGDSGGSSGGSKRTA<br>DGSEFESPKKKRKVSGGSSGGSTLNIEDEYRLHETSKEPDVSLGSTWLSDFPQAWAETGGMGLAVR<br>QAPLIIPLKATSTPVSIKQYPMSQEARLGIKPHIQRLLDQGILVPCQSPWNTPLLPVKKPGTNDYRPVQ<br>DLREVNKRVEDIHPTVPNPYNLLSGLPPSHQWYTVLDLKDAFFCLRLHPTSQPLFAFEWRDPEMGIS<br>GQLTWTRLPQGFKNSPTLFNEALHRDLADFRIQHPDLILLQYVDDLLLAATSELDCQQGTRALLQTL<br>GNLGYRASAKKAQICQKQVKYLGYLLKEGQRWLTEARKETVMGQPTPKTPRQLREFLGKAGFCRL<br>FIPGFAEMAAPLYPLTKPGTLFNWGPDQQKAYQEIKQALLTAPALGLPDLTKPFELFVDEKQGYAKG<br>VLTQKLGPWRRPVAYLSKKLDPVAAGWPPCLRMVAAIAVLTKDAGKLTMGQPLVILAPHAVEALV<br>KQPPDRWLSNARMTHYQALLLDTDRVQFGPVVALNPATLLPSGGSKRTADGSEFESPKKKRKVGSG<br>PAAKRVKLD* |

**Uncropped gel scan:**

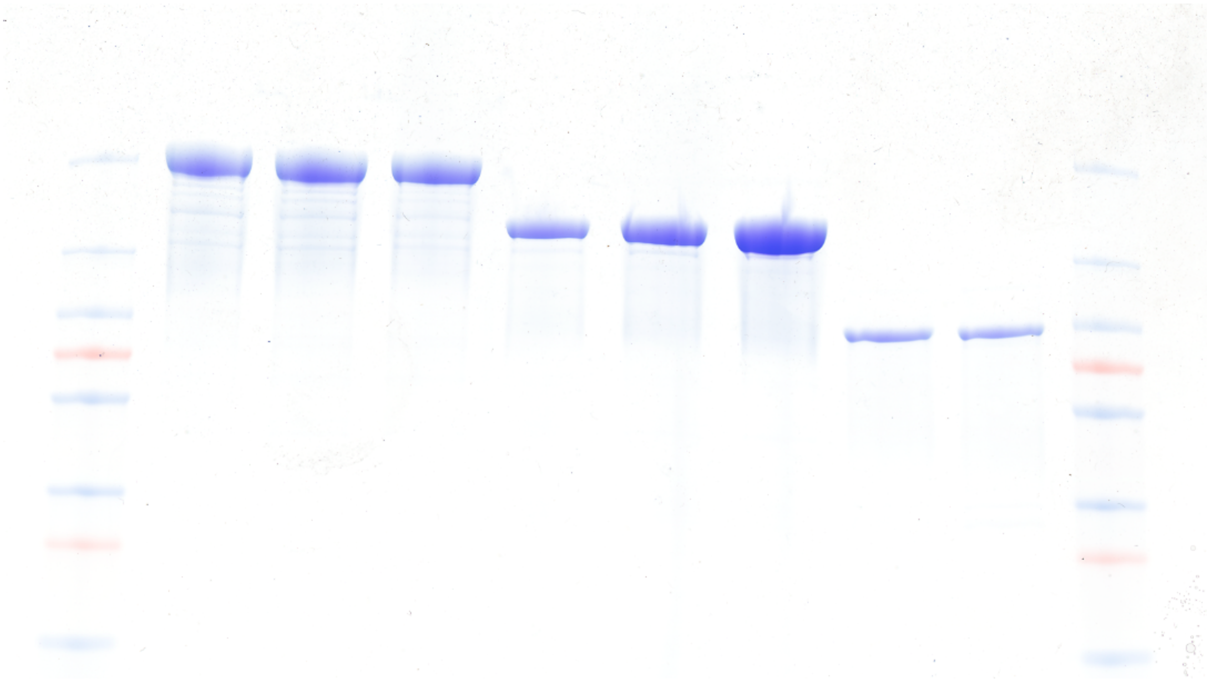

**Supplementary References:**


1. XuLi, LiuYakun & HanRenzhi. BEAT: A Python Program to Quantify Base Editing from Sanger Sequencing. *https://home.liebertpub.com/crispr* **2**, 223–229 (2019).
2. Böck, D. *et al.* In vivo prime editing of a metabolic liver disease in mice. *Sci Transl Med* **14**, 9238 (2022).
